# A Cyclin Dependent Kinase Regulatory Subunit (CKS) Gene of Pigeonpea Imparts Abiotic Stress Tolerance and Regulates Plant Growth and Development in *Arabidopsis*

**DOI:** 10.3389/fpls.2017.00165

**Published:** 2017-02-10

**Authors:** Srinath Tamirisa, Dashavantha R. Vudem, Venkateswara R. Khareedu

**Affiliations:** Centre for Plant Molecular Biology, Osmania UniversityHyderabad, India

**Keywords:** abiotic stress tolerance, abscisic acid, *Cajanus cajan*, cyclin-dependent kinase regulatory sub unit, glutathione, growth, development

## Abstract

Frequent climatic changes in conjunction with other extreme environmental factors are known to affect growth, development and productivity of diverse crop plants. Pigeonpea, a major grain legume of the semiarid tropics, endowed with an excellent deep-root system, is known as one of the important drought tolerant crop plants. Cyclin dependent kinases (CDKs) are core cell cycle regulators and play important role in different aspects of plant growth and development. The cyclin-dependent kinase regulatory subunit gene (*CKS*) was isolated from the cDNA library of pigeonpea plants subjected to drought stress. Pigeonpea *CKS* (*CcCKS*) gene expression was detected in both the root and leaf tissues of pigeonpea and was upregulated by polyethylene glycol (PEG), mannitol, NaCl and abscisic acid (ABA) treatments. The overexpression of *CcCKS* gene in *Arabidopsis* significantly enhanced tolerance of transgenics to drought and salt stresses as evidenced by different physiological parameters. Under stress conditions, transgenics showed higher biomass, decreased rate of water loss, decreased MDA levels, higher free proline contents, and glutathione levels. Moreover, under stress conditions transgenics exhibited lower stomatal conductance, lower transpiration, and higher photosynthetic rates. However, under normal conditions, *CcCKS*-transgenics displayed decreased plant growth rate, increased cell size and decreased stomatal number compared to those of wild-type plants. Real-time polymerase chain reaction revealed that *Cc*CKS could regulate the expression of both ABA-dependent and ABA-independent genes associated with abiotic stress tolerance as well as plant growth and development. As such, the *CcCKS* seems promising and might serve as a potential candidate gene for enhancing the abiotic stress tolerance of crop plants.

## Introduction

Plants often encounter extreme temperatures, drought, and high salt concentrations in soil, which are the major abiotic stresses that negatively affect plant growth and development (Krasensky and Jonak, 2012). A prerequisite for plants to acclimatize under extreme conditions is their ability to perceive stress signals and transduce them through a chain of signaling molecules, which ultimately interact with the regulatory elements of different stress-inducible genes and bring about changes in cellular gene expression (Kasuga et al., 1999; Tong et al., 2007; Tamirisa et al., 2014). An understanding of the mechanisms that regulate the expression of stress-responsive genes is essential for enhancing plant productivity under adverse environmental conditions (Priyanka et al., 2010a,b).

The development of plant organs directly depends on the frequency of cell division, parameters of the cell cycle, and number and size of the cells. These developmental processes are controlled by the molecular machinery that regulates cell cycle progression in coordination with nutritional, hormonal, developmental and environmental signals (Inze and Veylder, 2006; Tank and Thaker, 2011). A central role in the regulation of the cell cycle is played by a group of proteins known as cyclin-dependent kinases (CDKs), a family of protein kinases that were indentified for their role in regulating the cell cycle. CDKs are core cell cycle regulators that are involved in various processes of plant growth and development (Ma et al., 2015) and have been implicated in additional cellular processes such as transcription and translation. A progressive transition through different phases of the cell cycle is controlled by a conserved mechanism based on the sequential formation and activation of complexes between CDKs and their activating cyclin (CYC) subunits (De Veylder et al., 2003). CDK subunit proteins (CKS) might act as both activators and inhibitors of CDK activity (De Veylder et al., 2001a). CKS regulate CDK activity by functioning as docking factors for the CDK/CYC substrate complexes (Boruc et al., 2010a). The *Schizosaccharomyces pombe SUC1* gene was identified in fusion yeast by its ability to rescue certain temperature-sensitive *cdc2* mutants (Hayles et al., 1986), and its homolog, the *Saccharomyces cerevisiae CKS1* gene was found to be a suppressor of *CDC28* mutants (Hadwiger et al., 1989). The *SUC1/CKS1* homolog was isolated from *Arabidopsis* using yeast two-hybrid screening and designated as *AtCKS1* (De Veylder et al., 1997). The overexpression of *At*CKS1 in *Arabidopsis* decreased the leaf size and reduced the root growth rate without any effect on endoreduplication (De Veylder et al., 2001a).

Under biotic and abiotic stress conditions, plant growth is adversely affected as a result of cell cycle inhibition due to a prolonged S phase and delayed entry into mitosis (Kitsios and Doonan, 2011). Though the molecular interactions linking the cell cycle machinery to the perception of stress signals are not fully understood, recent studies have indicated the involvement of CDKs in the plant stress responses. An *Arabidopsis* CYCH;1 gene was reported to be involved in drought stress response by regulating the blue light-mediated stomatal opening as well as by controlling the reactive oxygen species (ROS) homeostasis (Zhou et al., 2013). However, their precise roles in abiotic stress responses are largely unknown (Kitsios and Doonan, 2011; Komaki and Sugimoto, 2012). Therefore, further research will help in the elucidation of possible mechanisms of CDK activity under different types of abiotic stresses.

In this study, we isolated the stress-responsive *Cajanus cajan* cyclin-dependent kinase regulatory subunit gene (*CcCKS*) from pigeonpea. The overexpression of *Cc*CKS in *Arabidopsis* conferred drought and salinity stress tolerance as well as marked changes in the growth and development of transgenic plants. This report clearly demonstrates that the *Cc*CKS in *Arabidopsis* affords abiotic stress tolerance and regulates plant growth and development.

## Materials and methods

### Plant materials and stress treatments

The pigeonpea seeds (variety ICP 8744) were disinfected with 0.1% (w/v) mercuric chloride and germinated in Petri plates. Four-week-old plants were subjected to polyethylene glycol (PEG) 6000 (20% w/v), mannitol (400 mM), NaCl (1.0 M), and ABA (100 μM) for 6 and 12 h. The sterilized seeds of *Arabidopsis* (Col-0), after stratification for 3 days at 4°C, were grown on Murashige and Skoog (1962) at 20 ± 1°C with a 16 h photoperiod under fluorescent light (120–150 μmol/m^2^ sec) in a Conviron growth chamber (Tamirisa et al., 2014).

### Subtraction cDNA library construction

Total RNA was isolated from 4-week-old 20% PEG-treated (−1.01 ± 0.02 Mpa) and unstressed (−0.49 ± 0.02 Mpa) plants by the guanidinium thiocyanate method (Sambrook and Russell, 2001). Poly (A+) RNA was purified from the total RNA through oligo (dT) cellulose chromatography using an mRNA isolation kit (Amersham Pharmacia Biotech, Asia Pacific Ltd, Quarry Bay, Hong Kong). The PCR select cDNA subtractive hybridization method was used to construct the cDNA library (Clontech, Mountain View, CA, USA) with 2 μg of mRNA isolated from control and PEG-treated plants (Tamirisa et al., 2014).

### Isolation of full-length *Cajanus cajan* cyclin-dependent kinase regulatory subunit gene (CcCKS)

Total RNA was isolated from the PEG (20%) treated 4-week-old plants as described above. RT-PCR for first-strand cDNA synthesis was carried out using a reaction mixture containing Tris-HCl (10 mM), KCl (50 mM), MgCl_2_ (1.5 mM), dNTPs (200 μM each), MMLV reverse transcriptase (2 units), total RNA (1.0 μg), and Oligo (dT) primer. The resultant cDNA product was used as a template in a PCR reaction using gene-specific primers to amplify the *CcCKS* gene. The full-length coding sequence (267 bp) of *CcCKS*, amplified using gene-specific primers and using the drought-stressed cDNA library as a template, was ligated at the *Sma*I-site of the pBSSK (+) vector and transformed into *E. coli* (TOP10) cells. The resultant recombinant clones were selected and confirmed by restriction digestion with *Xho*I and *Xba*I enzymes and later sequenced using an automated DNA sequencer. Homology search of nucleotide and amino acid sequences was carried out using BLAST (NCBI). A maximum likelihood phylogenetic tree was constructed based on the amino acid sequences of *Cc*CKS protein, and other CKS proteins from different plant species, using Clustal Omega and MEGA6 software. The bootstrap parameter was set at 100.

### Plant expression cassettes construction and transformation of *A. thaliana*

Plasmid DNA containing the *CcCKS* gene was digested with *Xho*I*-Xba*I enzymes and was subcloned into the pRT100 vector at the 3′ end of the CaMV35S promoter. Furthermore, the gene cassette was digested with *Hind*III and cloned into the pBI101 vector. The pBI101 vector containing *CcCKS* and *npt*II expression units and the pBI101 vector with *npt*II (empty vector) were mobilized independently into the EHA105 strain of *Agrobacterium* through triparental mating (Wise et al., 2006). The *Agrobacterium*-mediated floral dip method was carried out to transform *A. thaliana* (Clough and Bent, 1998). Seeds from the transformed plants were germinated on MS medium supplemented with kanamycin (50 mg L^−1^) to select the putative transformants.

### Molecular analysis of *CcCKS* transgenic *Arabidopsis* plants

Genomic DNA isolated from kanamycin tolerant plants was used to carry out PCR analysis. DNA isolated from untransformed plants (WT) was used as a negative control and plasmid (pBI12135S*npt*II-p35S*CcCKS*) DNA was used as a positive control. For PCR, plasmid DNA (10 ng) and genomic DNA (50 ng) were used as templates in separate reactions. The reactions were carried out using *CcCKS* gene-specific primers, and the amplified PCR products were analyzed on a 1.0% agarose gel.

### Southern blot analysis of *Arabidopsis* transgenic plants

Genomic DNA (15 μg) was isolated from three *CcCKS* transgenic plants by the CTAB method (Saghai-Maroof et al., 1984), and was digested independently with the *Hind*III enzyme. The digested DNA was resolved on 0.8% agarose gel and transferred to a positively charged nylon membrane as per the manufacturer's instructions (Amersham Pharmacia Biotech Asia Pacific Ltd, Quarry Bay, Hong Kong). A Southern blot analysis was performed according to Sambrook and Russell (2001). The *nptII* coding region was labeled with the AlkPhos Direct Labeling and Detection System according to the manufacturer's instructions (Amersham Pharmacia Biotech Asia Pacific Ltd, Quarry Bay, Hong Kong) and used as a probe.

### RT-PCR analysis *CcCKS* transgenic *Arabidopsis* plants

Total RNA was extracted from transgenic (CS1, CS2 and CS3), wild-type (WT) and vector control (VT) plants using the TRIZOL (Invitrogen, Carlsbad, CA, USA). RT-PCR was carried out as described earlier, and the amplified PCR products were analyzed on 1.0% agarose gel.

### Construction of the *CcCKS:GFP* fusion gene construct and generation of transgenic *Arabidopsis* plants

The *CcCKS* coding sequence without a termination codon was fused with the 5′ region of green fluorescent protein (*GFP*) and the fusion gene was driven by the CaMV35S promoter. The expression cassettes of pCaMV35S–*CcCKS*:*GFP/*pCaMV35S:*GFP* were subcloned into pBI101 containing the *npt*II expression unit and mobilized into the *Agrobacterium* (*EHA105* strain) through triparental mating. The transformed seedlings were selected on MS medium supplemented with kanamycin (50 mgL^−1^). Callus was induced from the roots of pCaMV35S:*GFP* and pCaMV35S–*CcCKS*:*GFP* transgenics on 2, 4-dichlorophenoxyacetic acid (2 mgL-1) containing medium. Individual callus cells were visualized under confocal microscopy (Leica SP5 Microsystems, Excitation: 488, and Emission: 520).

### Functional analysis of transgenics for abiotic stress tolerance

The seeds of WT, VT and homozygous transgenic (T_3_) *Arabidopsis* plants of CS1, CS2 and CS3 were surface-sterilized, placed on MS salt medium, and allowed to grow as described above. Drought and salt tolerance of 2-week-old transgenic seedlings was tested by growing them on MS medium supplemented with mannitol (150 mM) or NaCl (125 mM) for 7 days. Later, the seedlings were transferred onto the MS salt medium and allowed to recover for 10 days under normal conditions. After 10 days of recovery, the survival rate, root length and biomass of the seedlings were recorded. Another set of stressed seedlings were transferred to the soil, grown for maturity and photographed. For drought tolerance assays, 3-week-old plants growing in pots were withheld from water for 2 weeks, and then rewatered for 1 week (Sekhar et al., 2010).

### Relative water content measurements

The relative water contents (RWC) of 4-week-old transgenic plants of CS1, CS2 and CS3 along with WT and VT plants was measured. Fresh weight loss was calculated relative to the initial plant weight. To measure the fresh weight, plants were weighed immediately and left in the growth chamber (20 ± 1°C) until there was no further weight loss (desiccated weight). Finally, the plants were dried for 24 h at 70°C and the dry weights were recorded. The relative water content of the samples was measured using the formula RWC (%) = (desiccation weight—dry weight)/(fresh weight–dry weight) × 100 (Mao et al., 2010).

### Osmotic potential analysis

Two-week-old plants of CS1, CS2, and CS3 along with WT and VT plants were selected and frozen with liquid nitrogen for 30 s, and preserved at −80°C. Sap was collected by squeezing the leaves using a micro pestle, and the osmotic potential of the sap was determined using a vapor pressure osmometer (Vapro, Model 5520; Wescor Inc., Logan, UT, USA; Mao et al., 2010).

### Cell membrane stability analysis

Ten-day-old seedlings grown on 1X MS medium were placed on filter paper saturated with NaCl (150 mM) solution. As soon as symptoms of stress appeared (appx. after 6 h) in WT and VT plants, the seedlings were removed, rinsed thoroughly and immersed in 20 ml of double distilled water at room temperature (20 ± 1°C). The initial conductivity of samples was recorded after 2 h using a conductivity meter (Model Sension 5; HACH Company, Loveland, CO, USA). Later, samples were boiled for 30 min and, cooled to room temperature, and the final conductivity was measured. CMS was calculated using the formula CMS (%) = 1—initial electrical conductivity/electrical conductivity after boiling × 100 (Mao et al., 2010).

### Measurement of the leaf chlorophyll content

Leaf disks of CS1, CS2, and CS3 transgenic lines along with WT and VT plants were floated independently on water (control), 150 mM mannitol and 125 mM NaCl solutions at room temperature (20 ± 1°C) for 72 h. Leaf disks were then used to measure the chlorophyll content spectrophotometrically as described by Tamirisa et al. (2014).

### Measurement of the proline content

Fifteen-day-old *CcCKS* transgenic lines along with WT and VT seedlings were exposed to 150 mM mannitol and 125 mM NaCl for 3 days. Plant tissues were homogenized in 3% aqueous sulfosalicylic acid and the homogenates were centrifuged at 1000 rpm for 5 min. equal volumes of glacial acetic acid and ninhydrin were added to the supernatant collected. The proline content was quantified as described by Bates et al. (1973).

### Measurement of the malonaldehyde (MDA) content

Tissue (50 mg) was collected from stressed and unstressed transgenics, WT and VT seedlings and homogenized with 2 ml of 0.1% (w/v) cold trichlorocactic acid (TCA) on ice. The homogenates were centrifuged at 14,000 × g for 10 min at 4°C, after which 250 μl of supernatant was mixed with 1.5 ml of TCA/thiobarbituric acid (0.25% thiobarbituric acid containing 10% TCA) reagent. The MDA content was estimated according to Shin et al. (2012).

### Detection of superoxide radicals in WT and transgenic plants

The nitroblue tetrazolium (NBT) staining method was used for super oxide detection. Three-week-old transgenic, WT and VT plants were used for NBT staining. Plants were germinated on MS basal medium and later transferred to medium containing 150 mM mannitol (drought stress) and 125 mM NaCl (salt stress) for 3 days, along with unstressed seedlings. The treated seedlings were vacuum-infiltrated with 0.1 mg/ml NBT in 25 mM HEPES buffer, pH 7.6. The samples were incubated in the dark for 2 h in a growth chamber and later stained samples were transferred to 80% ethanol and incubated at 70°C for 10 min to remove chlorophyll. For each treatment, 20 seedlings of each line were used.

### Effect of ABA on seed germination and seedling growth

Seeds of transgenic and WT plants were placed on MS medium containing different concentrations of ABA (0–2.0 μM) to record the germination frequency. Further, germinated seedlings were allowed to grow for 20 days to assess their sensitivity to ABA.

### Measurement of stomatal aperture sizes

Rosette leaves of 3-week-old plants were detached and floated (abaxial side down) on solution containing 10 mM MES-KOH (pH 6.15), 30 mM KCl and 1 mM CaCl_2_ and were incubated under light for 2 h. Later, they were treated with ABA-containing solution for 2 h. In each treatment, 50 stomatal aperture sizes were measured under confocal microscopy.

### Root growth rate

For root growth rate analysis, the seeds were germinated on MS medium and placed vertically in the growth chamber. The position of the root tip was marked everyday on the backside of the Petri plates to determine the daily growth. After 20 days, the plates were scanned using a WinRHIZO standard systems desktop optical scanner. The daily length increase over the entire growing period was determined by measuring the distance between successive marks along the root axis using the WinRHIZO software. By dividing the daily growth by the precise time interval between the corresponding marks, the average growth rate was calculated (De Veylder et al., 2001a).

### Flow cytometric analysis

The DNA content was estimated by flow cytometric analysis. Plant materials (50 mg) of trangenic and WT plants were taken and chopped using a razor blade into 1 ml of ice-cold Otto I buffer in a Petri plates. The solution with the chopped plant tissue (suspension) was filtered through 40 μm nylon mesh; later, nuclei were pelleted by centrifugation (150 g/5 min). The nuclei were resuspended by gentle shaking and 100 μl of fresh Otto I buffer was added. The samples were incubated for 60 min. After incubation, Otto II buffer (1 ml) which includes propidium iodide (50 μg/ml) and RNase (50 μg/ml) was added. The samples were left at room temperature and analyzed after 20 min using a Guava EasyCyte flow cytometer (Millipore, France; Dolezel and Bartos, 2005).

### Pollen staining

Pollen staining was performed using the pollen collected from newly dehiscent flowers as described by Alexander (1969).

### Measurement of the glutathione (GSH) content

The GSH levels were measured in the intact roots and guard cells of *Arabidopsis* after conjugation with monochlorobimane (MCB), to produce a fluorescent glutathione S-bimane (GSB) adduct. Seedlings that were treated with 150 mM mannitol for 2 days were carefully removed from the agarose surface, and the roots were placed in a drop of 100 μM monochlorobimane. After labeling for 15 min, the roots were washed briefly in distilled water and mounted on a microscope slide with a cover slip. To measure the GSH levels in the guard cells, excised leaves from treated plants were incubated in a staining solution containing 100 μM of MCB for 2 h at room temperature. Photographs were taken using a fluorescent microscope (Olympus BX41fluorescence microscope). The captured images were used to measure the number of pixels/ fluorescence intensity using ImageJ software.

### Histological analysis

The cell density, stomatal density and stomatal index were determined as described by De Veylder et al. (2001a,b). Leaves of the same age and same relative position were sampled for analysis, and the leaf surface imprint method was used as described by Yu et al. (2008). Three leaves were sampled from each plant, and 10 of each WT, VT and transgenic plants were sampled.

### Scanning electron microscopy

Cotyledons from 3-week-old seedlings were processed and analyzed using scanning electron microscopy, as described De Veylder et al. (2002). Seedlings were fixed overnight in 4% paraformaldehyde and 1% glutaraldehyde in 0.1 M phosphate buffer (pH 7.2), followed by post-fixation in 2% osmiumtetroxide, and a graded ethanol series. These seedlings were mounted on stubs, sputter-coated with gold and examined using a scanning electron microscope (Zeiss, Germany).

### Measurement of stomatal conductance, transpiration, photosynthetic rates, and water use efficiency

The stomatal conductance, transpiration and photosynthetic rates were measured using a portable photosynthesis system (Li-Cor LI-6400XT). Four-week-old plants growing on soil were treated separately with 150 mM mannitol and 125 mM NaCl for 3 days. All of the photosynthetic measurements were taken at a constant air flow rate of 500 mmol s^−1^. Furthermore, the concentration of CO_2_ (400 mmol mol^−1^ using the system's CO_2_ injector), temperature (22°C), and photosynthetic photon flux density (1,200 mmol (photon) m^−2^ s^−1^) were maintained. Water use efficiency was calculated using the photosynthetic and transpiration measurements. Three measurements were taken on each plant, and 10 plants from WT, VT and each transgenic line were used for measurements.

### Quantitative real time PCR (qRT-PCR)

qRT-PCR was performed using RNA isolated from stressed and unstressed pigeonpea plants as described earlier. RNA was isolated from WT and transgenic plants subjected to mannitol (150 mM) stress for 2 days as well as from unstressed plants as described earlier. First strand cDNA was synthesized from RNA samples and used as a template for qRT-PCR. qRT-PCR analysis was performed using SYBR green master mix with the Applied Biosystems 7500 Real Time PCR system (USA). The genes were selected based on the literature available and the specific primers were designed. Each reaction was performed thrice, and the relative expression ratio was calculated using 2^−ΔΔct^ method with actin as the reference gene. The primers that were used for qRT-PCR are listed in Supplementary Table 1 (Tamirisa et al., 2014).

### Statistical analysis

All the experiments were done in triplicate using 20 seedlings in each treatment. Fresh tissue was collected for all the experiments. Mean, standard error and *t*-test were computed with the help of pre-loaded software in Excel, for statistical calculations.

## Results

### Characterization of the *CcCKS* gene

A cDNA clone of 267 bp sequence (KU522130), encoding a polypeptide of 88 amino acids, was designated as the cyclin-dependent kinase regulatory subunit gene (*CKS*). Phylogenetic tree analysis revealed that the *Cc*CKS protein was clustered in the same monophyletic group as putative CKS proteins from *Glycine max, Medicago truncatula* and *Arabidopsis lyrata* (Supplementary Figure S1). Further, the *Cc*CKS protein revealed high similarity with CDKs regulatory subunits of *G. max* (99%), *M. truncatula* (97%), *A. lyrata* (95%), *Nicotiana tomentosiformis* (95%), *Zea mays* (90%), *A. thaliana* (90%), and *Brassica rapa* (87%). To examine the subcellular localization of the *C*cCKS protein, pCaMV35S*-CcCKS:GFP* fusion and pCaMV35S*:GFP* gene (control) constructs were introduced into *Arabidopsis* and the individual callus cells were visualized under laser-scanning confocal microscopy. Cells expressing the *Cc*CKS:GFP fusion protein showed the fluorescence both in the nucleus and the cytoplasm (Supplementary Figure S2).

### Expression profile of *CcCKS* under abiotic stress conditions in pigeonpea

**Figure 1:**
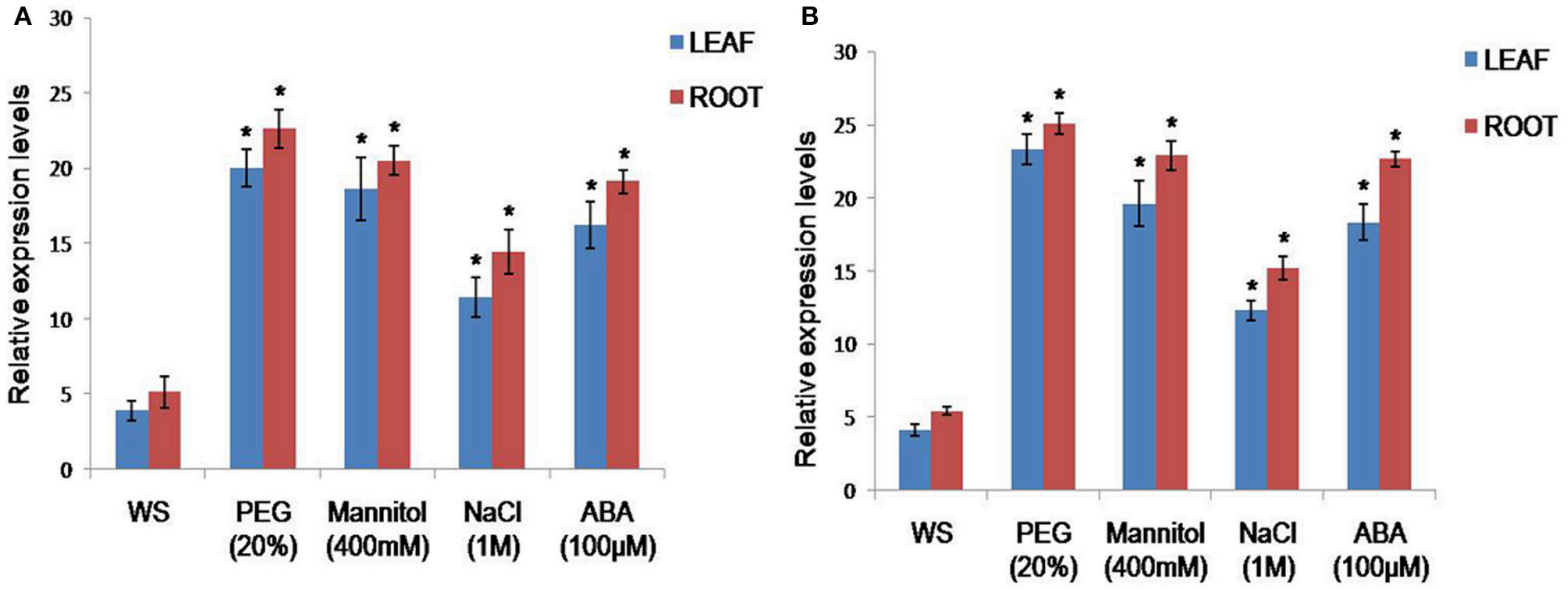
**Analysis of ***Cajanus cajan*** cyclin dependent kinase regulatory subunit gene (***CcCKS***) expression in pigeonpea under different treatment conditions by qRT- PCR**. Comparison of the relative transcript levels of *CcCKS* gene under (PEG) 6000 (20% w/v), Mannitol (400 mM), NaCl (1.0 M), and ABA (100 μM) stress for 6 h **(A)** and 12 h **(B)**. Actin has been used as reference gene. The vertical column indicates the relative transcript level. Bar represents mean and error bars represents SE from three independent experiments. Asterisks indicate significant differences in comparison with the WT at *P* < 0.05. WS represents without stress.

To analyse the stress-inducible nature of the *CcCKS* gene, real-time PCR was done using the total RNA isolated from pigeonpea plants after treatment under different stress conditions. These results revealed that increased levels of *CcCKS* transcripts were detected in both the roots and leaves of PEG-, mannitol-, NaCl- , and ABA- treated plants compared to untreated plants (Figure 1).

### Development of *CcCKS-*transgenic plants of *A. thaliana*

**Figure 2:**
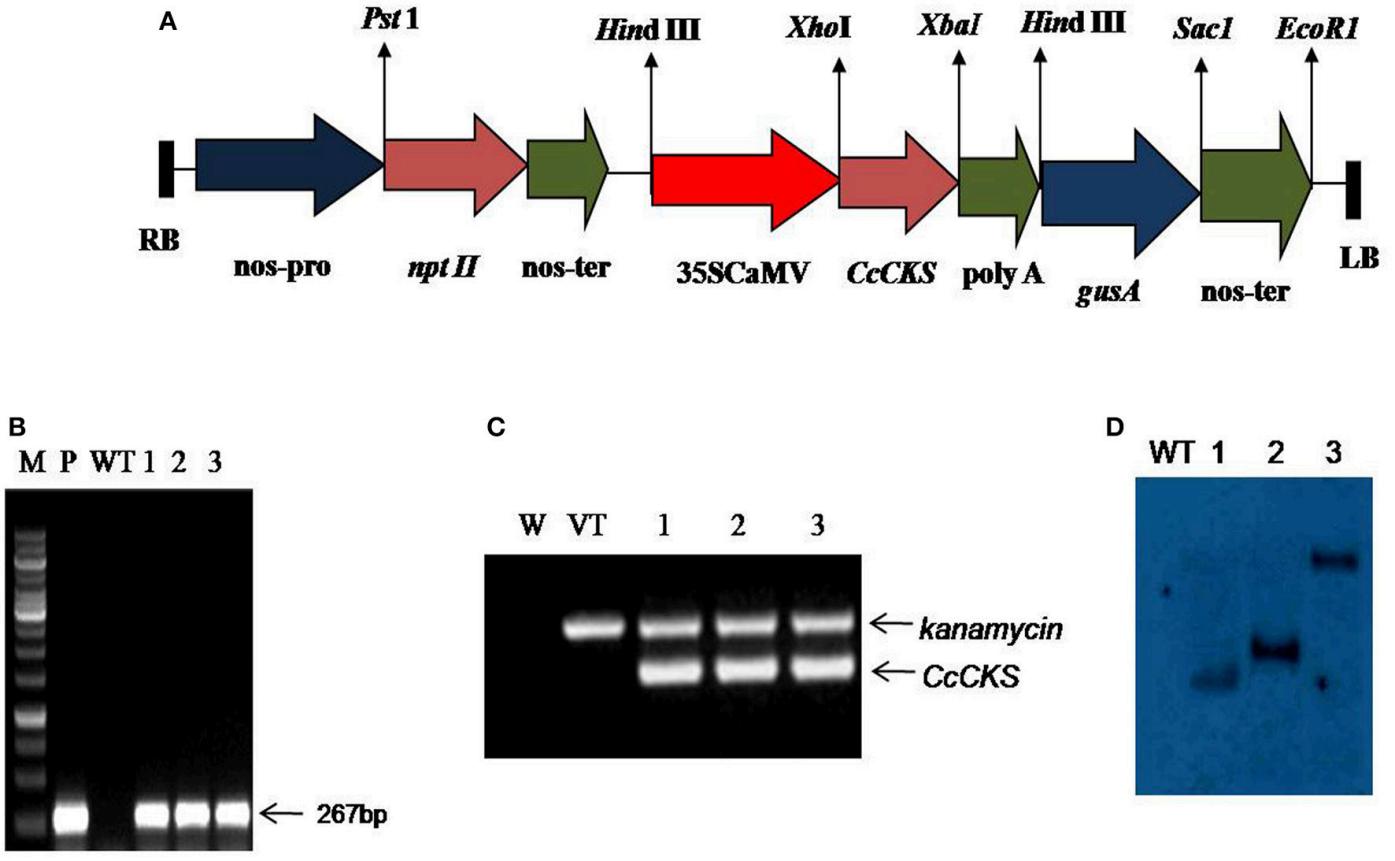
**T-DNA region of pBI101 containing ***CcCKS*** expression cassette and molecular confirmation of ***CcCKS*** transgenic ***Arabidopsis*** plants**. **(A)** Restriction map of T-DNA region of pBI101 containing *CcCKS* expression unit driven by CaMV35S promoter and *nptII* selection marker. **(B)** PCR analysis for the presence of *CcCKS* gene in the genomic DNA of transgenic *Arabidopsis plants*. Lane M: 1.0 kb DNA size standard marker, Lane P, Positive control; Lane WT, wild type; Lanes 1–3, PCR amplification of *CcCKS* from transgenic plants. **(C)** RT PCR analysis showing the expression of *kanamycin* and *CcCKS* genes in transgenic *Arabidopsis*. Lane WT, wild-type; Lane VT, vector control; Lanes 1, 2, and 3: different transgenic lines. **(D)** Southern blot analysis of transgenic *Arabidopsis* plants carrying *CcCKS* gene. Lane WT, wild type plant; Lanes 1, 2, and 3: transgenic plants of three different lines (CS1, CS2, and CS3).

Putative transgenic plants, obtained with plasmid constructs containing *CcCKS* (Figure 2A), were selected on MS medium containing kanamycin (50 mg L^−1^). PCR using *CcCKS* gene-specific primers amplified an ~250 bp fragment from the genomic DNA of transgenic plants, while no such band was observed in WT (Figure 2B). RT-PCR revealed the presence of *CcCKS* transcripts in pCaMV35S-*CcCKS* lines (Figure 2C). A Southern blot analysis of the genomic DNA isolated from transgenic lines demonstrated the existence of the transgene in the genome of *Arabidopsis*, when probed with *nptII* (Figure 2D). Homozygous transgenic lines of CS1, CS2 and CS3, containing a single T-DNA insertion, were chosen for subsequent experiments.

### Functional analysis of *CcCKS*-transgenic lines for abiotic stress tolerance

**Figure 3:**
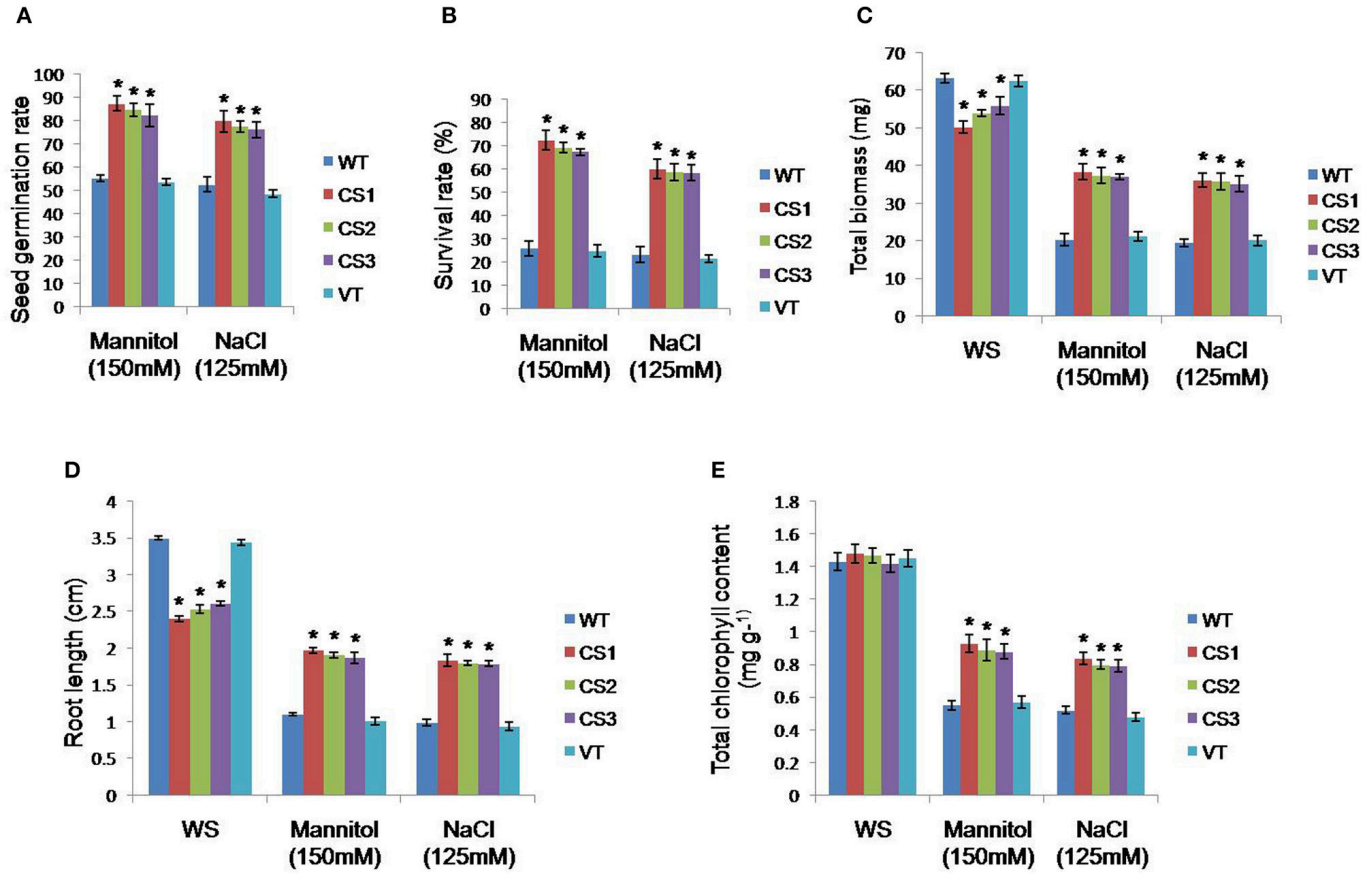
**Evaluation of ***CcCKS***-transgenics against different abiotic stresses**. Seed germination rate **(A)** of WT, VT, and transgenic plants under normal, 150 mM mannitol and 125 mM NaCl stress. Two-week-old seedlings of WT, VT, and transgenics were grown on 150 mM mannitol and 125 mM NaCl for 7 days and were later allowed to recover on MS plates. Data on survival rate **(B)**, total biomass **(C)** and root length **(D)** were recorded after 10 days of recovery. In each treatment, 20 seedlings of WT, VT, and two transgenic lines were used. Chlorophyll content **(E)** was determined from the leaf disks of control and transgenics after 72 h of incubation in water, 150 mM mannitol and 125 mM NaCl solutions independently at room temperature (20 ± 1°C). Bar represents mean and error bars represents SE from three independent experiments. Asterisks indicate significant differences in comparison with the WT at *P* < 0.05. WS, without stress; CS1, CS2, and CS3: three different transgenic lines; WT, wild type; VT, vector transformed plants.

No variation was observed in the seed germination of transgenic, VT or WT seeds under normal conditions. However, under mannitol (150 mM) and NaCl (125 mM) stress, higher germination rates were found in transgenic plants than in WT or VT plants (Figure 3A). To characterize the stress tolerance ability of transgenic plants, 2-week-old seedlings were subjected to drought (150 mM mannitol) and salt (125 mM NaCl) stress treatments for 7 days along with VT and WT seedlings. Transgenic lines of CS1, CS2, and CS3 exhibited increased survival rate, biomass and root lengths compared to VT and WT plants (Figures 3A–D).

**Figure 4:**
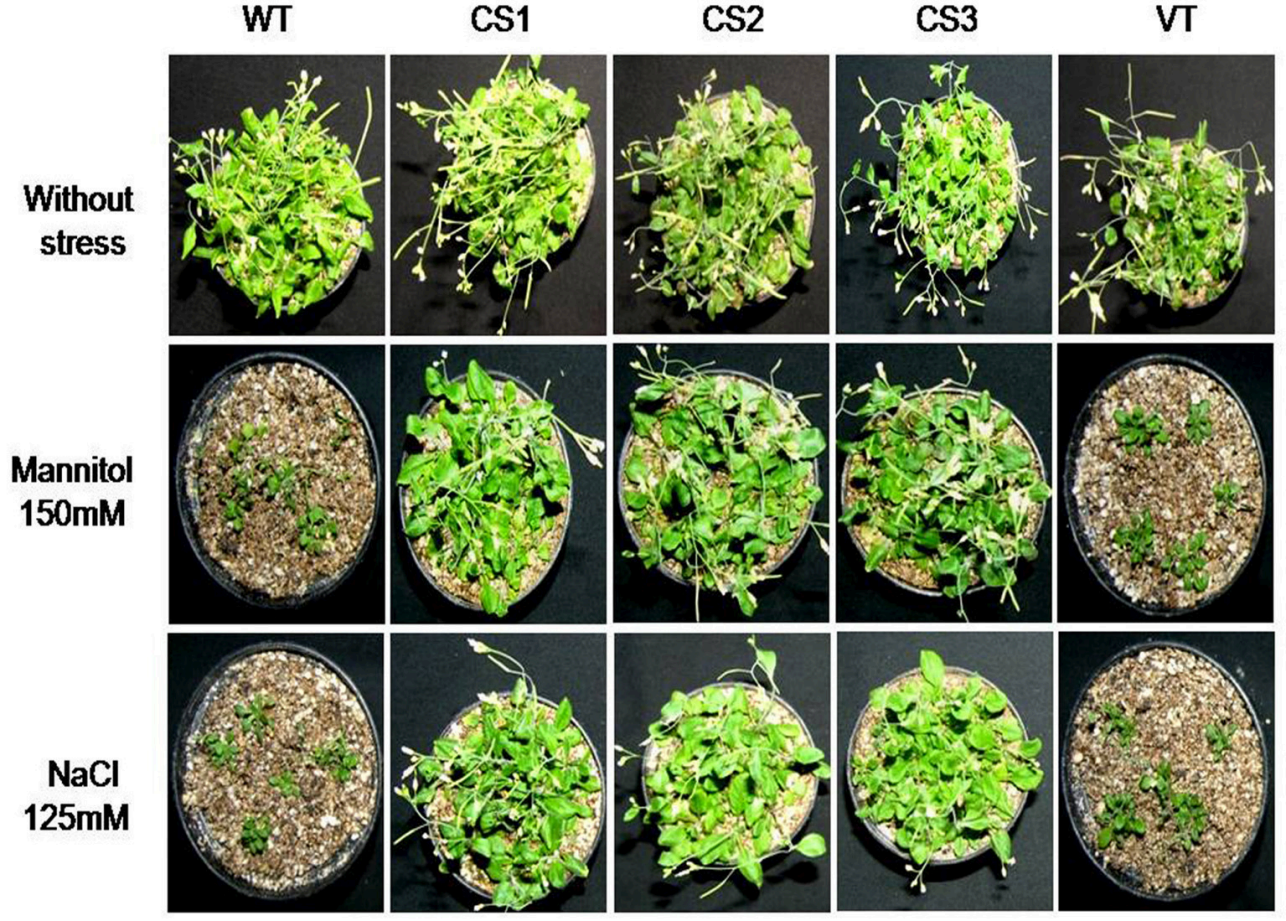
**Evaluation of transgenic plants expressing ***CcCKS*** gene against different abiotic stress conditions**. Two-week-old seedlings of WT, VT, and *Cc*CKS-transgenics were subjected to 150 mM mannitol, 125 mM NaCl for 7 days. In each treatment 20 seedlings were used. Treated seedlings were allowed to recover for 7 days at 20 ± 1°C temperature. Later, seedlings were transferred to soil and allowed to grow for 3 weeks under normal conditions, and were photographed. WT, wild type; VT, vector transformed; CS1, CS2, and CS3: three different transgenic lines.

Under normal conditions, no significant differences in chlorophyll contents were observed between the control and transgenic plants. The transgenic plants showed higher mean chlorophyll contents under both mannitol and NaCl stresses compared to those of WT and VT plants (Figure 3E). Further, the transgenic plants expressing *CcCKS* gene could successfully complete their reproductive cycle, while the control plants failed to reach the reproductive phase under drought and salt stress conditions (Figure 4). The transgenic plants subjected to low water conditions also showed greater tolerance compared to the WT and VT plants (Supplementary Figure S3A). Although water losses were observed in transgenic and control plants, the final relative water content of transgenics was significantly higher than that of WT and VT plants (Supplementary Figure S3B). When the osmotic potential of transgenic, VT, and WT plants, grown under well-watered conditions was measured, the CS1, CS2, and CS3 lines showed higher osmotic potential than in VT and WT plants (Supplementary Figure S3C).

### Estimation of the proline, malonaldehyde (MDA), and glutathione levels and the cell membrane stability in *CcCKS*-transgenic plants

**Figure 5:**
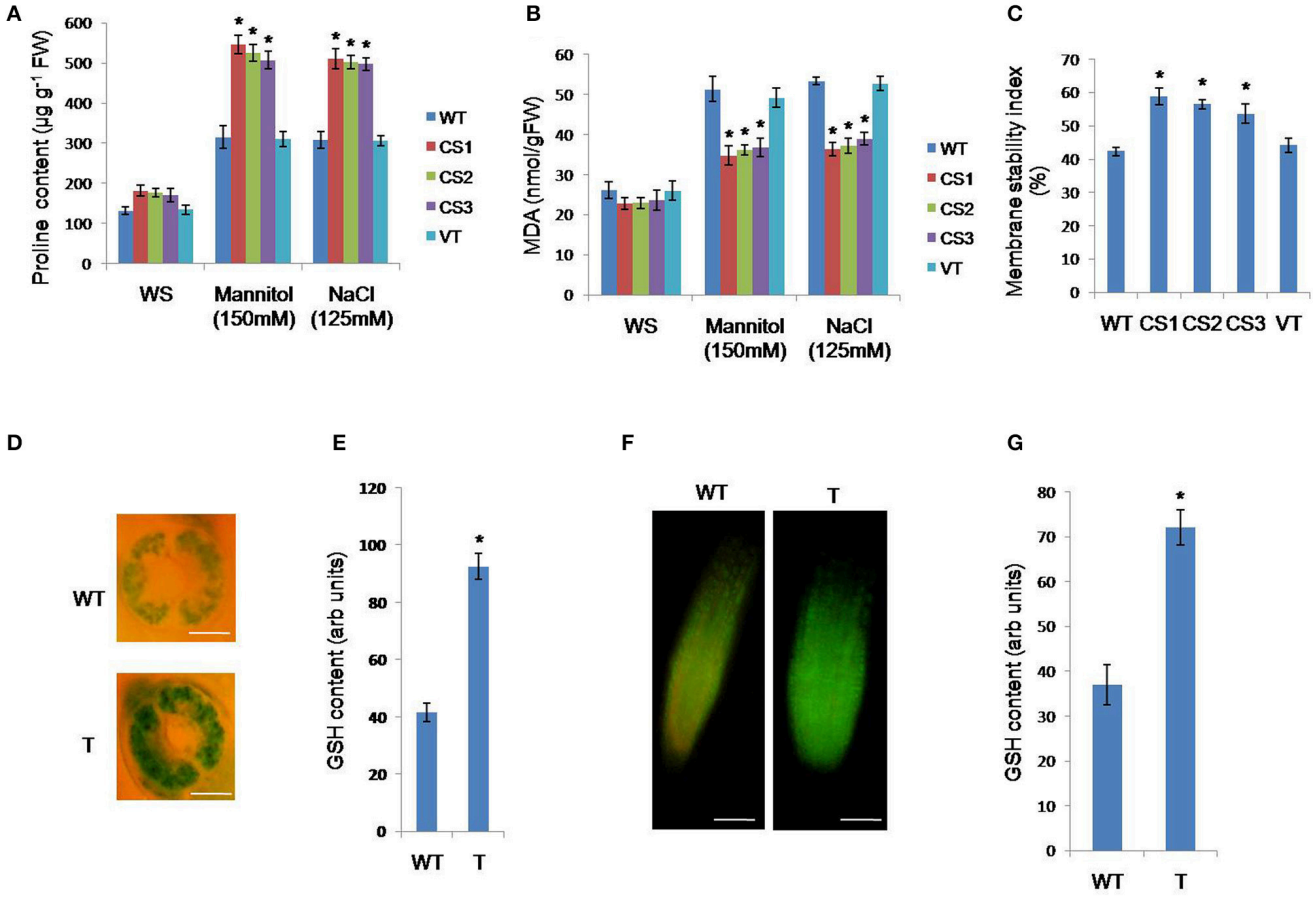
**Measurement of proline, MDA, osmotic potential, cell membrane stability and relative water contents of transgenic plants**. Two-week-old seedlings of WT, VT, and transgenics were grown on 150 mM mannitol for 3 days, for estimation of proline **(A)**, MDA **(B)**. Cell membrane stability **(C)** of transgenics, WT and VT seedlings were treated with 150 mM NaCl. Two-week-old *A. thaliana* seedlings, grown on MS medium, were subjected to mannitol (150 mM) for 2 days. GSH content in guard cells **(D,E)** and root meristem **(F,G)** of WT and transgenic plant. T represents *CcCKS-*transgenic and WT represents wild-type. Bar represents mean, and error bars represents SE from three independent experiments. For each treatment, 20 seedlings were used. Asterisks indicate significant differences in comparison with the WT at *P* < 0.05. WT, wild type; VT, vector transformed; CS1, CS2, and CS3: three different transgenic lines. **(D)** Scale bar 50 μm; **(F)**. Scale bar 100 μm.

To understand the physiological basis of stress tolerance of *CcCKS*-transgenic plants, the proline content was estimated under normal and stress conditions. Under normal conditions, transgenic lines showed enhanced proline levels, while there was no significant difference between WT and VT plants. When 15-day-old transgenics were subjected to mannitol (150 mM) and NaCl (125 mM) stresses, they could accumulate higher proline contents (Figure 5A), whereas, WT and VT plants showed lower levels of proline under similar stress conditions (Figure 5A).

The MDA contents in transgenic plants was significantly lower than those of WT and VT plants when subjected to mannitol and NaCl stress conditions (Figure 5B). However, there was no significant difference between WT and VT plants under similar stress conditions (Figure 5B).

To analyze the cell membrane stability, transgenic, WT, and VT plants were treated with NaCl (150 mM). Transgenic plants showed significantly higher membrane stability than that of WT and VT plants. However, there was no difference between WT and VT plants under similar stress conditions (Figure 5C).

Monochlorobimane (MCB) staining was performed to measure the GSH contents in the roots and guard cells of *Arabidopsis*. Under stress conditions, the fluorescence intensity of GSH in the guard cells of transgenic plants was much higher than that of WT plants (Figures 5D,E). Similarly, GSH fluorescence intensity in the roots of transgenic plants was higher than that of WT plants (Figures 5F,G).

### Detection of reactive oxygen species by NBT staining

**Figure 6:**
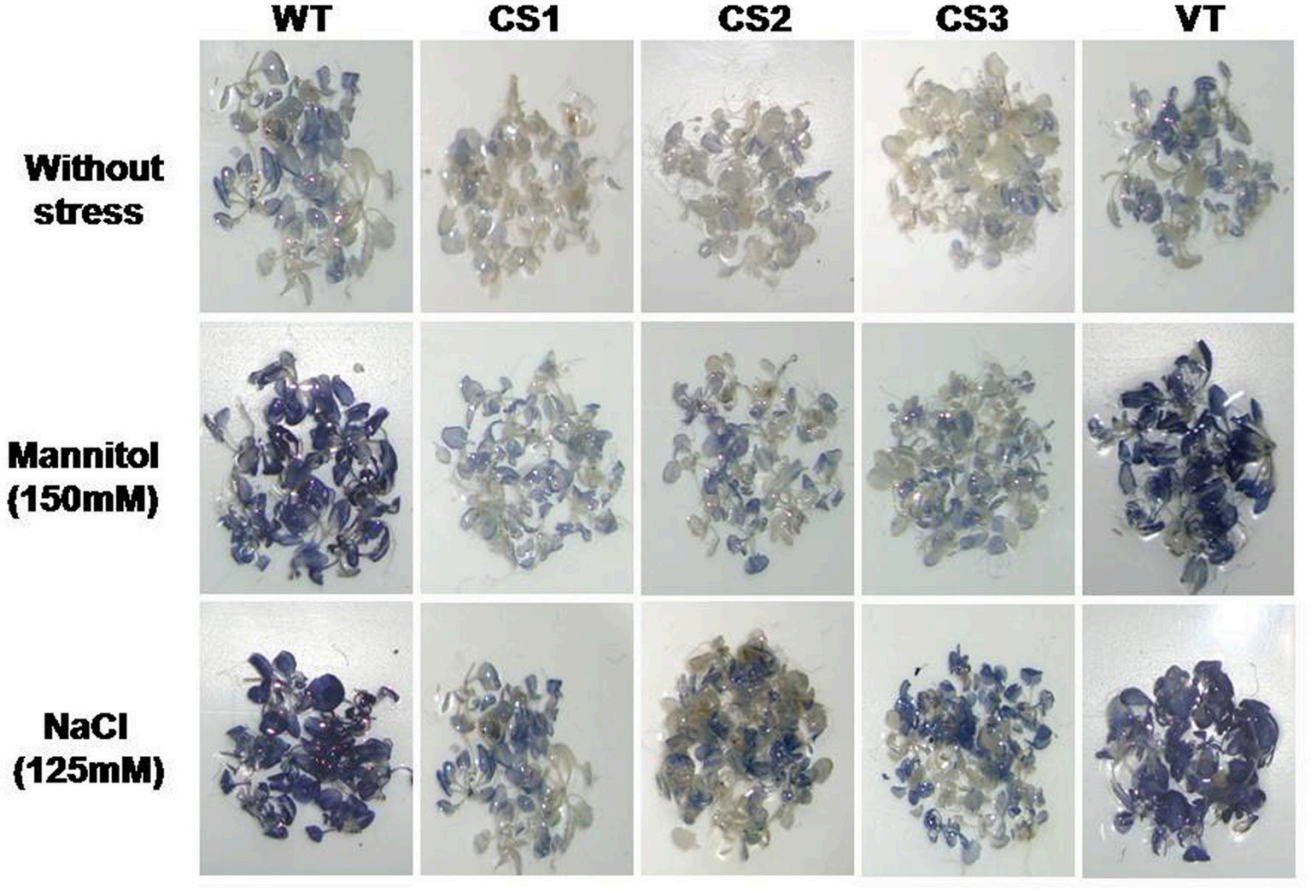
**Superoxide detection in transgenic plants subjected to different abiotic stresses**. Two-week-old *A. thaliana* seedlings grown on MS medium were subjected to mannitol (150 mM) and NaCl (125 mM) for 72 h. For each treatment, 20 seedlings were used. NBT staining was done for superoxide detection. WT: wild type; VT: vector control; CS1, CS2, and CS3: three different transgenic lines.

The superoxide concentration in the 2-week-old seedlings, treated with mannitol (150 mM) and NaCl (125 mM), was examined using nitroblue tetrazolium (NBT) staining. Under unstressed conditions, transgenic and control seedlings revealed a low intensity of staining. However, under stress conditions, the transgenics exhibited a lower staining intensity than that of WT and VT seedlings, as visualized by the purple formazan deposit (Figure 6).

### ABA sensitivity of *CcCKS*-transgenic plants and their stomatal aperture sizes

The seed germination rate of transgenic plants was severely inhibited by ABA compared with the WT plants (Supplementary Figure S4A). Transgenics when treated with ABA, showed a greater reduction in the stomatal aperture size compared to that of WT plants (Supplementary Figure S4B). Furthermore, transgenic seedlings showed greater hypersensitivity to ABA than that of WT plants (Supplementary Figure S4C).

### Stomatal conductance, transpiration, photosynthetic rates, and water use efficiency under stress treatments

**Figure 7:**
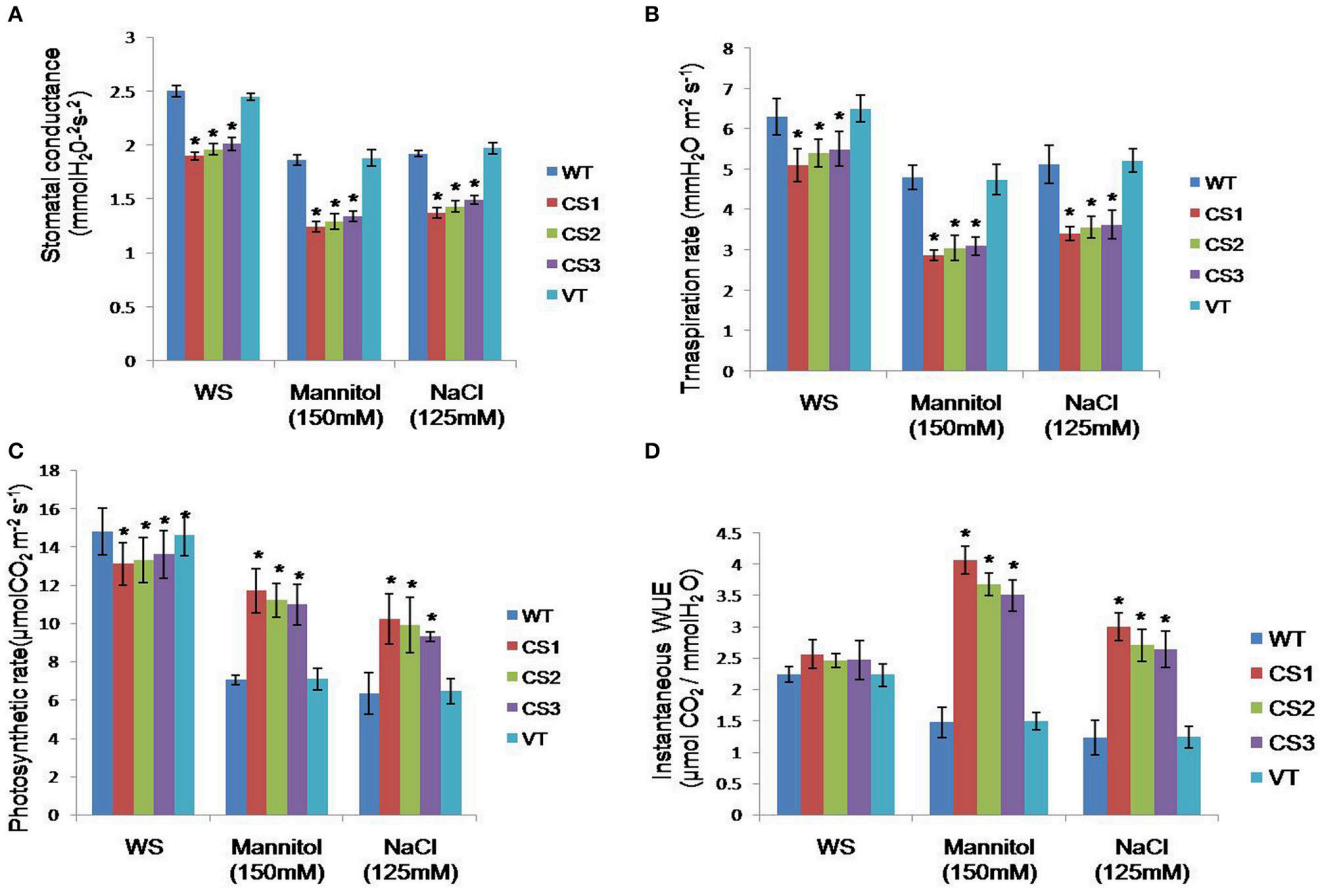
**Measurement of stomatal conductance, photosynthesis, transpiration and water use efficiency of transgenic plants**. Four weeks old plants grown on soil were treated with 150 mM mannitol (drought stress) and 125 mM NaCl (salt stress) separately for 3 days. Stomatal conductance **(A)**, transpiration **(B)**, photosynthetic rates **(C)** and water use efficiency **(D)** were measured using a portable photosynthesis system (Li-Cor LI-6400XT). Three measurements were made on each plant, and 10 plants from WT as well as each transgenic line were used for measurements. WT represents wild type; VT represents vector transformed; CS1, CS2, and CS3 represent transgenic lines. Bar represents mean error bars represents SE from three independent experiments. Asterisks indicate significant differences in comparison with the WT at *P* < 0.05.

Under normal conditions, the transgenic lines showed lower stomatal conductance, transpiration and photosynthetic rates compared to WT plants. However, transgenics subjected to mannitol (150 mM) and NaCl (125 mM) stresses, exhibited lower stomatal conductance, lower transpiration, higher photosynthetic rates and water use efficiency. By contrast, WT and VT plants showed higher stomatal conductance, greater transpiration and lower photosynthetic rates under similar stress conditions (Figures 7A–D).

### Pollen viability

Fertile pollen grains were observed in both transgenic and WT plants without any differences in the pollen fertility as evidenced by Alexander staining (Supplementary Figure S5A).

### Flow cytometric analysis

To determine whether the cell cycle was affected in the *Cc*CKS overexpressing plants, flow cytometric analysis was performed on the nuclei isolated from the roots of WT and transgenic plants. Wild-type roots of *Arabidopsis* showed the typical patterned peaks ranging from 2C to 8C, and the C values of transgenic lines were similar to those of WT plants (Supplementary Figure S5B).

### Developmental changes in *CcCKS* transgenics in the root growth rate, leaf size, pavement cell number, stomatal number, stomatal index, and stomatal morphology

**Figure 8:**
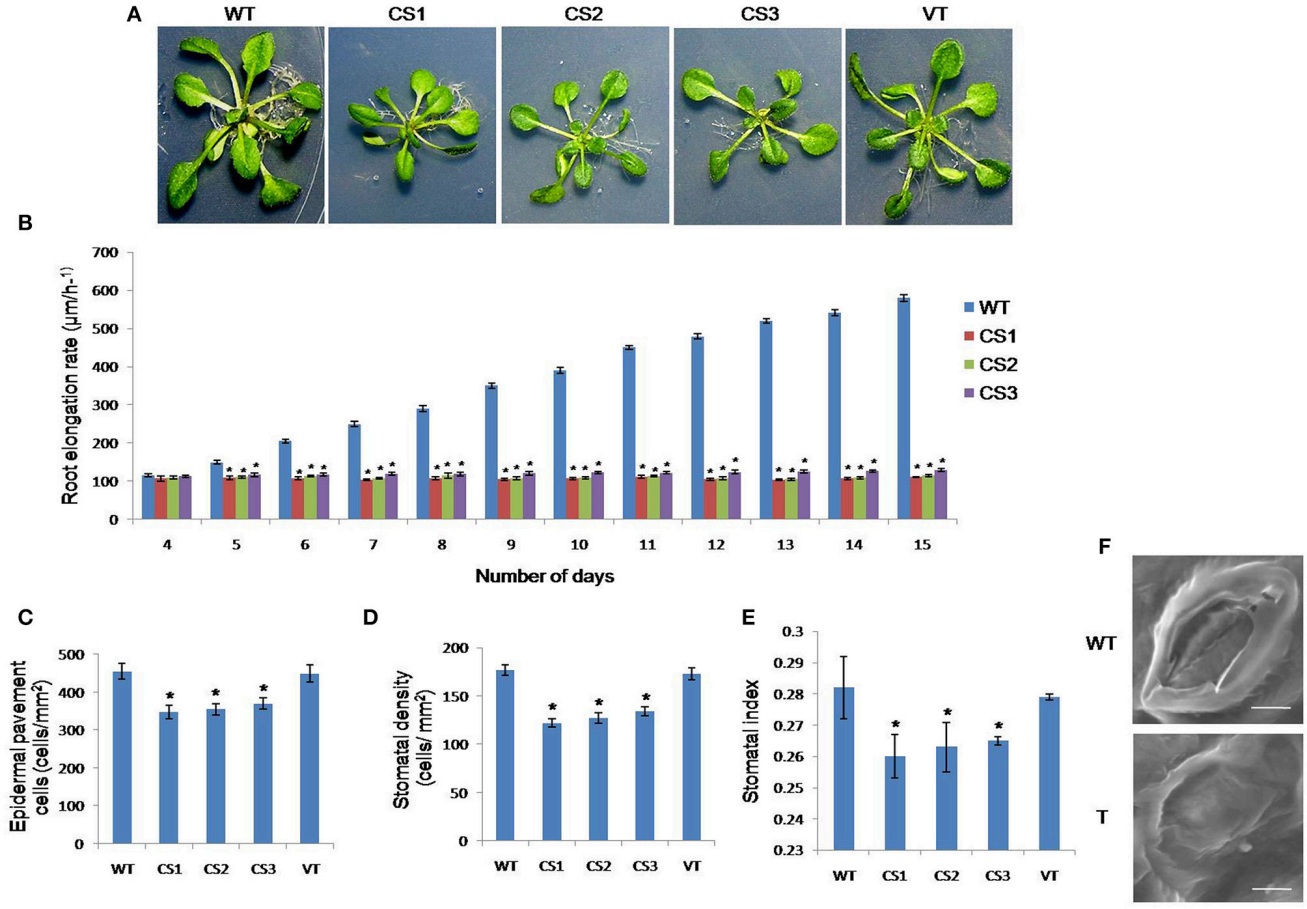
**Effect of ***CcCKS*** on growth and development of transgenic ***Arabiodpsis*** plants. (A)** Rosette sizes of three weeks old WT and *CcCKS* independent transgenic lines grown on MS medium. **(B)** Primary root elongation rates of transgenic lines and WT plants. **(C)** Density of epidermal pavement cells, **(D)** Stomatal density, **(E)** Stomatal index, **(F)** Scanning electron microscope images of normal (WT) and aberrant stomatal cells in transgenic (T). WT: wild type; CS1, CS2, and CS3: 35S transgenic lines. Bar represents mean error bars represents SE from three independent experiments. Asterisks indicate significant differences in comparison with the WT at *P* < 0.05. Scale (F) bar 2μm.

Three weeks after seed germination, WT, VT, and *CcCKS* transgenic plants exhibited distinct observable differences in their morphological features. Transgenic plants showed smaller rosettes and leaves compared to WT plants (Figure 8A). There were no observable differences in the root length of transgenic and WT seedlings during the initial 3 days of root growth. Later, *CcCKS* transgenic lines showed an approximately steady growth rate, while WT plants showed accelerated root growth during the 15 day growth period analyzed (Figure 8B). The pavement cell density on the adaxial surface in similar leaf areas from CS1, CS2, and CS3 transgenics was lower than that of WT and VT plants (Figure 8C). The size of cells leaving the meristem was greater in *CcCKS* transgenics (85 ± 7.87 μm) than in WT (47 ± 3.54 μm). The results clearly showed significant differences between transgenics and WT plants (*P* < 0.05). Moreover, the mature cell length in the transgenic roots was slightly longer (191 ± 8.96 μm) than in WT cells (175 ± 10.1 μm).

Compared with WT and VT plants, transgenics revealed a substantially lower stomatal density and stomatal index (Figures 8D,E). The morphology of stomata in WT and *CcCKS*-transgenic *Arabidopsis* plants appeared normal. However, scanning electron micrographs clearly demonstrated a few (3%) abnormal stomata in the transgenic plants (Figure 8F).

### Analysis of expression levels of selected genes in *CcCKS*-transgenic plants by qRT-PCR

**Figure 9:**
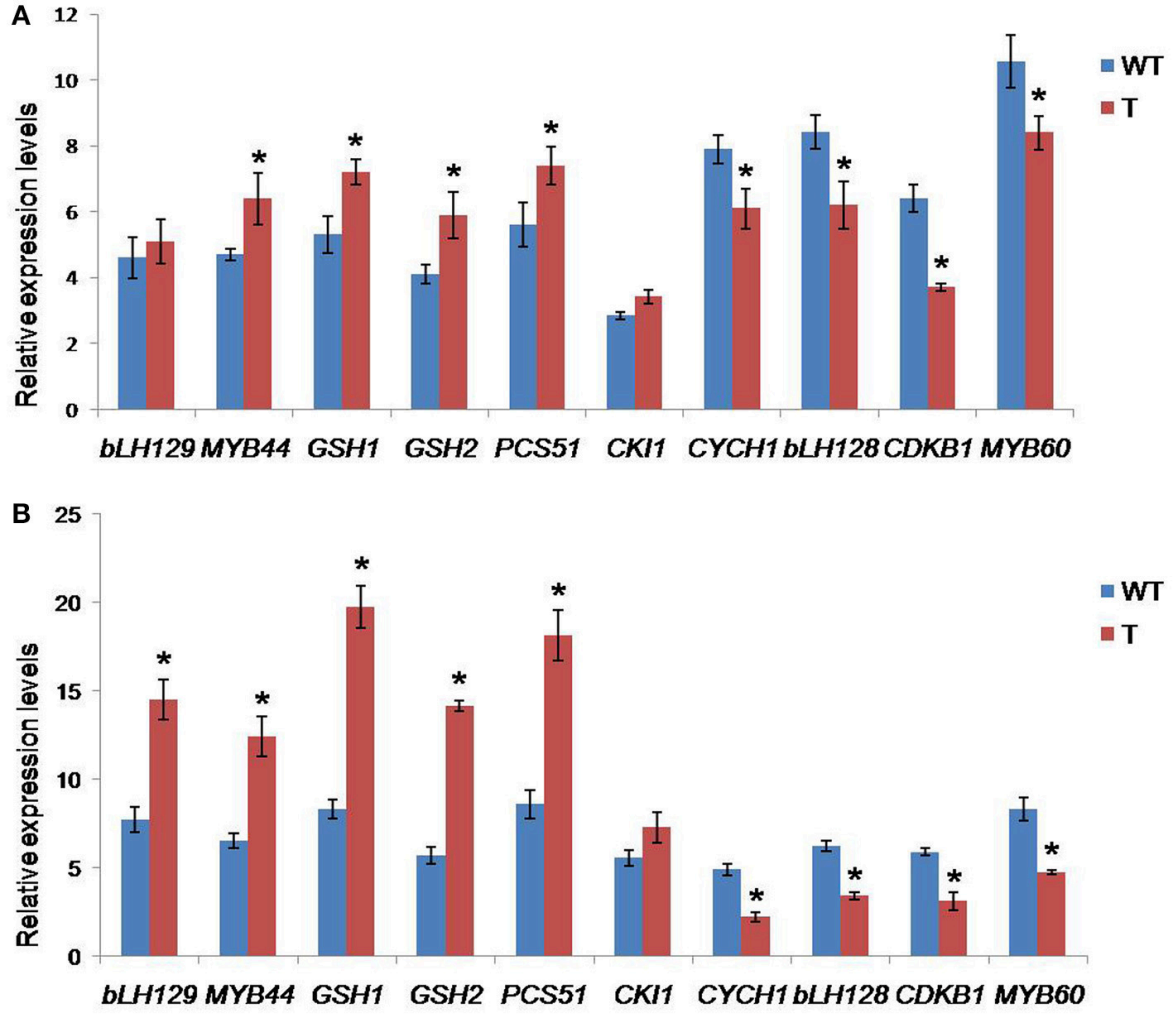
**qRT-PCR analysis for the expression profiles of selected genes in wild type and transgenic ***Arabidopsis*** under drought stress**. Comparisons of the relative transcript levels of different genes in *CcCKS-*transgenic (T) and wild-type (WT) plants under normal **(A)** and 150 mM mannitol **(B)** stress conditions. Actin was used as a reference gene. The vertical column indicates the relative transcript level. Bar represents mean and error bars represents SE from three independent experiments. Asterisks indicate significant differences in comparison with the WT at *P* < 0.05.

Under stress conditions, real time PCR analysis revealed substantial increases in the expression levels of certain genes, viz., delta-1-pyrroline 5-carboxylase synthetase (P5C1), bHLH129 transcription factor, MYB44 transcription factor, glutathione synthetase (*gsh2*), and glutamate cysteine ligase (*gsh1*), in transgenic plants compared to those of WT plants. However, the genes encoding cyclin H1, cyclin-dependent kinase B1, MYB60 transcription factor and bHLH128 transcription factor were significantly downregulated in transgenics under similar stress conditions compared to WT plants (Figure 9).

## Discussion

A stress-responsive cyclin-dependent kinase regulatory subunit gene (*CKS*) was isolated from the cDNA library constructed using the RNA isolated from drought stressed pigeonpea plants. The *Cc*CKS protein structure analysis indicated its similarity to other CKS proteins of *G. max, M. truncatula, A. lyrata, Z. mays, N. tomentosiformis*, and *B. rapa*.

Higher levels of *CcCKS* expression were detected both in the leaves and roots under drought, ABA and salt stress treatments, compared to unstressed plants, indicating the stress-responsive nature of the gene. It was previously reported that the maize cyclin-dependent kinase regulatory subunit gene (*ZmCKS2*) showed variable expression levels in different parts of the seedlings, and was upregulated by drought and MeJA treatments and downregulated by cold or ABA treatments (Bao et al., 2010).

The localization of the *Cc*CKS:GFP fusion protein in both the nucleus and the cytoplasm (Supplementary Figure S2) suggests that the CcCKS protein can enter the nucleus and it might interact with different proteins to regulate the expression of various genes involved in the plant development and stress response. *Arabidopsis* CKS1 and CKS2 subunits were highly and homogenously expressed in the nucleus and cytoplasm (Boruc et al., 2010b).

Under normal conditions, *CcCKS* transgenic plants were smaller in size with a significant reduction in fresh weight, in addition to smaller rosettes and leaves. Transgenics also exhibited fewer epidermal cells per unit leaf area (Figure 8). In transgenics, the cells leaving the root meristem were larger than that of WT plants. Moreover, the mature cells in the transgenic roots were longer than in WT plants. The difference in plant growth rate observed in transgenics compared to WT might be due to the fewer cells being produced and not because of the inhibition of cell expansion. In yeast, the expression of *Arabidopsis* the cyclin-dependent kinase regulatory subunit gene (*AtCKS1*) inhibited cell division due to its binding with CDKs (De Veylder et al., 1997). Transgenic *Arabidopsis* plants overexpressing *AtCKS1* showed reduced leaf size and root growth rates. Moreover, changes in both cell cycle duration and meristem size strongly affected the root elongation rate in transgenic plants (De Veylder et al., 2001a). The overexpression of the CDK inhibitor gene (*ICK1*) resulted in fewer numbers of cells in transgenic *Arabidopsis* plants, and the cells were larger than those of WT plants (Wang et al., 2000).

A flow cytometric analysis of *CcCKS*-transgenic plants demonstrated the absence of any observable effects in ploidy levels. A well coordinated progression of mitotic and endoreduplication cycles is essential for normal plant growth, development and their response to environmental changes (Komaki and Sugimoto, 2012). Furthermore, no differences were observed in the pollen fertility between transgenic and WT plants. These results strongly suggest that *CcCKS* overexpression did not affect male fertility or endoreduplication in the transgenic lines.

The *CcCKS*-transgenic plants showed higher seed germination, survival rates, biomass, root growth, and chlorophyll content under drought and salt stress conditions. Furthermore, they could grow to maturity and set seed while the WT and VT plants failed to reach the flowering stage (Figures 3, 4, Supplementary Figure S3). These results clearly demonstrate that the transgenics were able to withstand drought and salinity treatments. *Arabidopsis cdkg2* mutants showed higher percentages of seed germination, seedling growth and root elongation than those of WT under NaCl stress conditions (Ma et al., 2015).

Increased levels of proline content and the upregulation of *P5CS1* gene involved in proline biosynthesis might have contributed to the osmotic potential adjustment, greater stress tolerance ability, and better growth of transgenics under stress conditions (Figure 5). Proline plays vital roles as a free radical scavenger, protecting macromolecules against denaturation, in cell-cycle control, and in differentiation and programmed cell death, in addition to modulating the expression of numerous genes that influence plant growth and development under normal conditions (Mattioli et al., 2009; Szabados and Savoure, 2010; Biancucci et al., 2015). Furthermore, proline deficiency downregulates major cyclin genes at the transcriptional level, causing cell cycle arrest and the suppression of cell proliferation (Wang et al., 2014). Lower levels of MDA and higher cell membrane stability and relative water content were observed in *CcCKS* transgenic lines under stress conditions (Figure 5). MDA is an indicator of lipid peroxidation due to the accumulation of ROS under stress condition (Davey et al., 2005). These results are further corroborated by lower levels of superoxide in the transgenic plants than in WT and VT plants as evidenced by the NBT staining (Figure 6).

Under stress conditions, higher levels of GSH as well as the upregulation of γ-glutamyl cysteine synthetase and glutathione synthetase genes involved in the biosynthesis of GSH were observed in transgenic plants compared to WT plants (Figure 5). Hence, the higher GSH levels might have resulted in the detoxification of ROS, leading to lower MDA levels in transgenic plants. Glutathione is the most important antioxidant in plants and is essential for defense against abiotic and biotic stresses. It also functions in redox-signaling, the modulation of defense gene expression and the regulation of enzymatic activities (Zechmann, 2014). Furthermore, the involvement GSH was reported in the regulation of cell division in the apical root meristem and in protecting proteins during seed dehydration (Sánchez-Fernández et al., 1997; Cairns et al., 2006). Transgenic *Arabidopsis* plants expressing tomato glutathione S-transferase (*Le*GSTU2) revealed enhanced resistance to NaCl and mannitol stresses (Xu et al., 2015).

Under stress conditions, the transgenics showed a significant reduction in the stomatal conductance, lower transpiration and higher photosynthetic rates compared to WT plants. In contrast, under normal conditions, the transgenics showed lower stomatal conductance, transpiration and photosynthetic rates compared to WT plants. In general, stomatal density affects water, and CO_2_ exchange. Decreased stomatal aperture results in a reduced transpiration rate in order to maintain the balance between water absorption and loss for better photosynthesis (Han et al., 2013).

Increased expression levels of *bHLH129* and decreased transcript levels of *bHLH128* were found in the transgenic plants. Basic helix-loop-helix (*bHLH*) transcription factors are one of the largest transcription factor families that regulate various aspects of plant growth and development, in addition to responses to abiotic stresses (Liu et al., 2014). Increased levels of *MYB44* transcripts were observed in transgenic plants than in WT plants. *MYB44* transgenic *Arabidopsis* plants efficiently prevented the accumulation of destructive ROS (Persak and Pitzschke, 2014).

In transgenic plants, lower levels of *CYCLIN H-1* and *MYB60* transcripts were found under stress conditions. In *Arabidopsis, CYCH;1* showed predominant expression in guard cells while its expression was downregulated by dehydration (Zhou et al., 2013). Furthermore, it regulated drought stress responses through an ABA-independent pathway (Zhou et al., 2013). Similarly, *MYB60* inhibited stomatal closure and root growth during drought stress and its ectopic expression in *Arabidopsis* resulted in greater susceptibility to the stress (Oh et al., 2011). Lower levels of cyclin-dependent kinase B1-1 (*CDKB1*) transcripts were observed in transgenic plants than in WT plants. A decrease in the stomatal number, stomatal index, and formation of abnormal stomata in transgenic plants might be attributed to the down-regulation of the *CDKB1* gene. *Arabidopsis CDKB1-1* gene was found highly expressed in guard cells and stomatal precursor cells, and thus played an important role in the stomatal development (Boudolf et al., 2004).

ABA mediates a variety of physiological processes including responses to drought, salt and cold stress (Zhu et al., 2007). In transgenic plants, the upregulation of gene transcripts involved in both ABA-dependent and ABA-independent pathways reveal the ability of *CcCKS* to regulate their expression. Some of the CDKs in plants play important roles in regulating the expression of genes by participating in the transcription process and by interacting with the other activators (Cui et al., 2007).

Transgenics were hypersensitive to ABA and had smaller stomatal apertures compared to WT plants treated with ABA. These results clearly indicate that *Cc*CKS functions in the ABA signal transduction pathway. Under stress conditions, the ABA level increases in plants and is known to the mediate transcriptional regulation of stress-responsive gene expression (Kang et al., 2002). ABA also associates the abiotic stress response with plant development since it appears to play a direct role in repressing and upregulating some cell cycle genes (Kitsios and Doonan, 2011).

In this study, we cloned a stress responsive *CcCKS* gene from the cDNA library of pigeonpea. The overexpression of *Cc*CKS in *Arabidopsis* caused enhanced tolerance to both drought and salinity stresses. The transgenic plants also showed marked changes in plant growth and development. An overview of the results suggests that *Cc*CKS possibly acts upstream of genes involved in stress tolerance as well as plant growth and development, and thus plays an important role in the crosstalk between the signaling pathways that integrate abiotic stress tolerance with growth and development.

## Author contributions

VK, DV, and ST: Conceived and designed the experiments; ST: Performed the experiments; ST, DV, and VK: Analyzed the data and wrote the paper.

### Conflict of interest statement

The authors declare that the research was conducted in the absence of any commercial or financial relationships that could be construed as a potential conflict of interest.

## Supplementary material

Supplementary Table 1 **Primers sequences used in the study**.

Supplementary Figure S1 **Phylogenetic tree of ***Cc***CKS protein**.

Supplementary Figure S2 **Expression cassettes of pCaMV35S-CcCKS-***GFP*** fusion gene and ***s***ubcellular localization of CcCKS-GFP fusion protein in ***Arabidopsis*** callus cells**.

Supplementary Figure S3 **Evaluation of ***Cc***CKS transgenics against water withhold conditions. (A)** Three weeks old plants growing in pots were withheld from water for two weeks, and then rewatered for one week. Photographs of plants were taken before drought stress, at the end of drought stress and after rewatering. WT represents wild type; *VI* represents vector transformed; CS1, CS2 and CS3 represent transgenic lines. **(B)** Comparison of relative water contents of detached rosettes of WT, *VI* and transgenic plants. **(C)** Measurement of osmotic potential of transgenic lines, WT and VT plants under normal and 150 mM mannitol.

Supplementary Figure S4 **Hypersensitivity response of wild type and ***CcCKS*** transgenic ***Arabidopsis*** to ABA. (A)** Germination rates of wild type (WT) and transgenic (T) *Arabidopsis* seedlings on ABA containing medium, **(B)** Stomatal aperture sizes of wild type (WT) and transgenic (T) *Arabidopsis* plants treated with ABA. **(C)** Morphology of wild type (WT) and transgenic (T) seedlings grown on ABA containing medium.

Supplementary Figure S5 **Pollen fertility and ploidy level in transgenic and wild type plants. (A)** Pollen fertility of wild type and *Cc*CKS transgenics plants. T represents *Cc*CKS-transgenic and WT represents wild-type. Pink color shows fertile pollen grains. **(B)** Ploidy levels in wild-type and *Cc*CKS transgenics. WT represents wild-type, CS1, CS2 and CS3 represent transgenic lines. (Scale bar 25μm).

## References

[B1] Alexander M. P. (1969). Differential staining of aborted and nonaborted pollen. Stain Technol. 44, 117–122. 10.3109/10520296909063335 4181665

[B2] Bao Z. Z. Min L. Hui Y. L. Deng F. Z. Ying H. L. Su Y. S. (2010). Isolation and expression analysis of drought-induced gene *ZmCKS2* in maize. Acta Agron. Sin. 36, 945–952. 10.1016/S1875-2780(09)60055-9

[B3] Bates L. S. Waldran R. Teare I. D. (1973). Rapid determination of free proline for water studies. Plant Soil 39, 205–208. 10.1007/BF00018060

[B4] Biancucci M. Mattioli R. Moubayidin L. Sabatini S. Costantino P. Trovato M. (2015). Proline affects the size of the root meristematic zone in *Arabidopsis*. BMC Plant Biol. 15:263. 10.1186/s12870-015-0637-8 26514776 PMC4625561

[B5] Boruc J. Mylle E. Duda M. De Clercq R. Rombauts S. Geelen D.. (2010b). Systematic localization of the *Arabidopsis* core cell cycle proteins reveals novel cell division complexes. Plant Physiol. 152, 553–565. 10.1104/pp.109.148643 20018602 PMC2815867

[B6] Boruc J. Van den Daele H. Hollunder J. Rombauts S. Mylle E. Hilson P.. (2010a). Functional modules in the *Arabidopsis* core cell cycle binary protein–protein interaction network. Plant Cell 22, 1264–1280. 10.1105/tpc.109.073635 20407024 PMC2879739

[B7] Boudolf V. Barrôco R. de Engler J. A. Verkest A. Beeckman T. Naudts M.. (2004). B1-type cyclin-dependent kinases are essential for the formation of stomatal complexes in *Arabidopsis thaliana*. Plant Cell 16, 945–955. 10.1105/tpc.021774 15031414 PMC412868

[B8] Cairns N. G. Pasternak M. Wachter A. Cobbett C. S. Meyer A. J. (2006). Maturation of *Arabidopsis* seeds is dependent on glutathione biosynthesis within the embryo. Plant Physiol. 141, 446–455. 10.1104/pp.106.077982 16531482 PMC1475471

[B9] Clough S. J. Bent A. F. (1998). Floral dip: a simplified method for *Agrobacterium*-mediated transformation of *Arabidopsis thaliana*. Plant J. 16, 735–743. 10.1046/j.1365-313x.1998.00343.x 10069079

[B10] Cui X. Fan B. Scholz J. Chen Z. (2007). Roles of *Arabidopsis* cyclin-dependent kinase C complexes in cauliflower mosaic virus infection, plant growth, and development. Plant Cell 19, 1388–1402. 10.1105/tpc.107.051375 17468259 PMC1913762

[B11] Davey M. Stals E. Panis B. Keulemans J. Swennen R. (2005). High-throughput determination of malondialdehyde in plant tissues. Anal. Biochem. 347, 201–207. 10.1016/j.ab.2005.09.041 16289006

[B12] De Veylder L. Beeckman T. Beemster G. T. de Almeida Engler J. Ormenese S. Maes S. (2002). Control of Proliferation, Endoreduplication and Differentiation by the *Arabidopsis* E2Fa/DPa transcription factor. EMBO J. 21, 1360–1368. 10.1093/emboj/21.6.1360 11889041 PMC125359

[B13] De Veylder L. Beeckman T. Beemster G. T. Krols L. Terras F. Landrieu I.. (2001b). Functional analysis of cyclin-dependent kinase inhibitors of *Arabidopsis*. Plant Cell 13, 1653–1668. 10.1105/tpc.13.7.1653 11449057 PMC139548

[B14] De Veylder L. Beemster G. T. Beeckman T. Inzé D. (2001a). CKS1At overexpression in *Arabidopsis thaliana* inhibits growth by reducing meristem size and inhibiting cell-cycle progression. Plant J. 25, 617–626. 10.1046/j.1365-313x.2001.00996.x 11319029

[B15] De Veylder L. Joubès J. Inzé D. (2003). Plant cell cycle transitions. Curr. Opin. Plant Biol. 6, 536–543. 10.1016/j.pbi.2003.09.001 14611951

[B16] De Veylder L. Segers G. Glab N. Casteels P. Van Montagu M. Inzé D. (1997). The *Arabidopsis* Cks1At protein binds to the cyclin-dependent kinases Cdc2aAt and Cdc2bAt. FEBS Lett. 412, 446–452. 10.1016/S0014-5793(97)00822-3 9276444

[B17] Dolezel J. Bartos J. (2005). Plant DNA flow cytometry and estimation of nuclear genome size. Ann. Bot. 95, 99–110. 10.1093/aob/mci005 15596459 PMC4246710

[B18] Hadwiger J. A. Wittenberg C. Mendenhall M. D. Reed S. I. (1989). The *Saccharomyces cerevisiae* CKS1 gene, a homolog of the *Schizosaccharomyces pombe* suc1+ gene, encodes a subunit of the Cdc28 protein kinase complex. Mol. Cell Biol. 9, 2034–2041. 10.1128/MCB.9.5.2034 2664468 PMC362996

[B19] Han X. Tang S. An Y. Zheng D. C. Xia X. L. Yin W. L. (2013). Overexpression of the poplar NF-YB7 transcription factor confers drought tolerance and improves water use efficiency in *Arabidopsis*. J. Exp. Bot. 64, 4589–4601. 10.1093/jxb/ert262 24006421 PMC3808328

[B20] Hayles J. Beach D. Durkacz B. Nurse P. (1986). The fission yeast cell cycle control gene *cdc2*: isolation of a sequence *suc1* that suppresses *cdc2* mutant function. Mol. Gen. Genet. 202, 291–293. 10.1007/BF00331653 3010051

[B21] Inze D. Veylder L. (2006). Cell cycle regulation in plant development Annu. Rev. Genet. 40, 77–105. 10.1146/annurev.genet.40.110405.090431 17094738

[B22] Kang J. Y. Choi H. I. Im M. Y. Kim S. Y. (2002). *Arabidopsis* basic leucine zipper proteins that mediate stress-responsive abscisic acid signaling. Plant Cell 14, 343–357. 10.1105/tpc.010362 11884679 PMC152917

[B23] Kasuga M. Liu Q. Miura S. Yamaguchi-Shinozaki K. Shinozaki K. (1999). Improving plant drought, salt, and freezing tolerance by gene transfer of a single stress-inducible transcription factor. Nat. Biotechnol. 17, 287–291. 10.1038/7036 10096298

[B24] Kitsios G. Doonan J. H. (2011). Cyclin dependent protein kinases and stress responses in plants. Plant Signal. Behav. 6, 204–209. 10.4161/psb.6.2.14835 21512322 PMC3121979

[B25] Komaki S. Sugimoto K. (2012). Control of the plant cell cycle by developmental and environmental cues Plant Cell Physiol. 53, 953–964. 10.1093/pcp/pcs070 22555815

[B26] Krasensky J. Jonak C. (2012). Drought, salt, and temperature stress-induced metabolic rearrangements and regulatory networks. J. Exp. Bot. 63, 1593–1608. 10.1093/jxb/err460 22291134 PMC4359903

[B27] Liu W. Tai H. Li S. Gao W. Zhang M. Xie C.. (2014). bHLH122 is important for drought and osmotic stress resistance in *Arabidopsis* and in the repression of ABA catabolism. New Phytol. 201, 1192–1204. 10.1111/nph.12607 24261563

[B28] Ma X. Qiao Z. Chen D. Yang W. Zhou R. Zhang W.. (2015). CYCLIN DEPENDENT KINASE G2 regulates salinity stress response and salt mediated flowering in *Arabidopsis thaliana*. Plant Mol. Biol. 88, 287–299. 10.1007/s11103-015-0324-z 25948280

[B29] Mao X. Zhang H. Tian S. Chang X. Jing R. (2010). TaSnRK2.4, an SNF1-type serine/threonine protein kinase of wheat (*Triticum aestivum* L.), confers enhanced multistress tolerance in *Arabidopsis*. J. Exp. Bot. 61, 683–696. 10.1093/jxb/erp331 20022921 PMC2814103

[B30] Mattioli R. Costantino P. Trovato M. (2009). Proline accumulation in plants: not only stress. Plant Signal Behav. 4, 1016–1018. 10.4161/psb.4.11.9797 20009553 PMC2819507

[B31] Murashige T. Skoog F. A. (1962). Revised medium for rapid growth and bioassays with tobacco tissue cultures. Physiol. Plant. 15, 473–497. 10.1111/j.1399-3054.1962.tb08052.x

[B32] Oh J. E. Kwon Y. Kim J. H. Noh H. Hong S. W. Lee H. (2011). A dual role for MYB60 in stomatal regulation and root growth of *Arabidopsis thaliana* under drought stress. Plant Mol. Biol. 77, 91–103. 10.1007/s11103-011-9796-7 21637967

[B33] Persak H. Pitzschke A. (2014). Dominant repression by *Arabidopsis* transcription factor MYB44 causes oxidative damage and hypersensitivity to abiotic stress. Int. J. Mol. Sci. 15, 2517–2537. 10.3390/ijms15022517 24531138 PMC3958865

[B34] Priyanka B. Sekhar K. Reddy V. D. Rao K. V. (2010a). Expression of pigeonpea hybrid-proline-rich protein encoding gene (CcHyPRP) in yeast and *Arabidopsis* affords multiple abiotic stress tolerance. Plant Biotechnol. J. 8, 76–87. 10.1111/j.1467-7652.2009.00467.x 20055960

[B35] Priyanka B. Sekhar K. Sunitha T. Reddy V. D. Rao K. V. (2010b). Characterization of expressed sequence tags (ESTs) of pigeonpea (*Cajanus cajan* L.) and functional validation of selected genes for abiotic stress tolerance in *Arabidopsis thaliana*. Mol. Genet. Genomics 283, 273–287. 10.1007/s00438-010-0516-9 20131066

[B36] Saghai-Maroof M. A. Soliman K. M. Jorgensen R. A. Allard R. W. (1984). Ribosomal DNA spacer-length polymorphisms in barley: mendelian inheritance, chromosomal location and population dynamics. Proc. Natl. Acad. Sci. U.S.A. 81, 8014–8019. 10.1073/pnas.81.24.8014 6096873 PMC392284

[B37] Sambrook J. Russell D. W. (2001). Molecular cloning: A Laboratory Manual. New York, NY: Cold Spring Harbor Laboratory Press.

[B38] Sánchez-Fernández R. Fricker M. Corben L. B. White N. S. Sheard N. Leaver C. J.. (1997). Cell proliferation and hair tip growth in the *Arabidopsis* root are under mechanistically different forms of redox control. Proc. Natl. Acad. Sci. U.S.A. 94, 2745–2750. 10.1073/pnas.94.6.2745 11038608 PMC20161

[B39] Sekhar K. Priyanka B. Reddy V. D. Rao K. V. (2010). Isolation and characterization of a pigeonpea cyclophilin (*CcCYP*) gene, and its over-expression in *Arabidopsis* confers multiple abiotic stress tolerance. Plant Cell Environ. 33, 1324–1338. 10.1111/j.1365-3040.2010.02151.x 20374537

[B40] Shin L. J. Lo J. C. Yeh K. C. (2012). Copper chaperone antioxidant protein1 is essential for copper homeostasis. Plant Physiol. 159, 1099–1110. 10.1104/pp.112.195974 22555879 PMC3387697

[B41] Szabados L. Savoure A. (2010). Proline: a multifunctional amino acid. Trends Plant Sci. 15, 89–97. 10.1016/j.tplants.2009.11.009 20036181

[B42] Tamirisa S. Vudem D. R. Khareedu V. R. (2014). Overexpression of pigeonpea stress-induced cold and drought regulatory gene (*CcCDR*) confers drought, salt, and cold tolerance in *Arabidopsis*. J. Exp. Bot. 65, 4769–4781. 10.1093/jxb/eru224 24868035 PMC4144763

[B43] Tank J. G. Thaker V. S. (2011). Cyclin dependent kinases and their role in regulation of plant cell cycle. Biol. Plantarum. 55, 201–212. 10.1007/s10535-011-0031-9

[B44] Tong S. Ni Z. Peng H. Dong G. Sun Q. (2007). Ectopic overexpression of wheat TaSrg6 gene confers water stress tolerance in *Arabidopsis*. Plant Sci. 172, 1079–1086. 10.1016/j.plantsci.2007.03.011

[B45] Wang G. Zhang J. Fan X. Sun X. Qin H. Xu N.. (2014). *Proline responding1* plays a critical role in regulating general protein synthesis and the cell cycle in maize. Plant Cell 26, 2582–2600. 10.1105/tpc.114.125559 24951479 PMC4114953

[B46] Wang H. Zhou Y. Gilmer S. Whitwill S. Fowke L. C. (2000). Expression of the plant cyclin dependent kinase inhibitor ICK1 affects cell division, plant growth and morphology. Plant J. 24, 613–623. 10.1046/j.1365-313x.2000.00899.x 11123800

[B47] Wise A. A. Lu Z. Binns A. N. (2006). Three methods for the introduction of foreign DNA into *Agrobacterium*. Methods Mol. Biol. 343, 43–53. 10.1385/1-59745-130-4:43 16988332

[B48] Xu J. Xing X. J. Tian Y. S. Peng R. H. Xue Y. Zhao W.. (2015). Transgenic *Arabidopsis* plants expressing tomato glutathione S-transferase showed enhanced resistance to salt and drought stress. PLoS ONE 10:e0136960. 10.1371/journal.pone.0136960 26327625 PMC4556630

[B49] Yu H. Chen X. Hong Y. Y. Wang Y. Xu P. Ke S. D.. (2008). Activated expression of an *Arabidopsis* HD-START protein confers drought tolerance with improved root system and reduced stomatal density. Plant Cell 20, 1134–1151. 10.1105/tpc.108.058263 18451323 PMC2390749

[B50] Zechmann B. (2014). Compartment specific importance of glutathione during abiotic and biotic stress. Front. Plant Sci. 5:566. 10.3389/fpls.2014.00566 25368627 PMC4202713

[B51] Zhou X. F. Jin Y. H. Yoo C. Y. Lin X. L. Kim W. Y. Yun D. J. (2013). CCYCLIN;1 regulated drought stress responses and blue light-induced stomatal opening by inhibiting reactive oxygen species accumulation in *Arabidopsis*. Plant Physiol. 162, 1030–1041. 10.1104/pp.113.215798 23656895 PMC3668038

[B52] Zhu S. Y. Yu X. C. Wang X. J. Zhao R. Li Y. Fan C. R.. (2007). Two calcium-dependent protein kinases, CPK_4_ and CPK_11_, regulate abscisic acid signal transduction in *Arabidopsis*. Plant Cell 19, 3019–3036. 10.1105/tpc.107.050666 17921317 PMC2174700

