# Supplementary material for: A Cyclin Dependent Kinase Regulatory Subunit (CKS) Gene of Pigeonpea Imparts Abiotic Stress Tolerance and Regulates Plant Growth and Development in Arabidopsis
Source: Front Plant Sci. 2017 Feb 10;8:165. doi: 10.3389/fpls.2017.00165 (PMC5301084; doi:10.3389/fpls.2017.00165)
Supplement: Supplementary Table 1 — Primers sequences used in the study. [file Table1.DOC]

**Supplementary Table 1. Primers sequence used in the study**

| **GENE NAME** | **PRIMER SEQIENCE** |
| --- | --- |
| CDK-F | 5’- GGCTCGAGATGGGTCAGATCCAGTACTC -3’ |
| CDK-R | 5’- GGTCTAGATCATTTGACAAGCATGCTTT-3’ |
| CDKF GFP-F | 5’- GG TCTAGAATGGGTCAGATCCAGTACTC -3’ |
| CDKF GFP-R | 5’- GGGGATCCTTTGACAAGCATGCTTTGCTGG -3’ |
| KAN-F | 5’- TGTTCCGGCTGTCAGCGCAG -3’ |
| KAN-R | 5’- GATCCTCGCCGTCGGGCATG -3’ |
| Cyclin H1-F (ID:At5g27620) | 5’- GGAGTGATTGACTTTGATAGG -3’ |
| Cyclin H1-R | 5’- CGT TTGGTGTATCATTGGAGC -3’ |
| CDK B1:1- F (ID: AT3G54180.1) | 5’- GCTCCTGAAGTTCTTCTTGGA -3’ |
| CDK B1:1 -R | 5’- TTCGGCTGGATTGTACTTGAG -3’ |
| CDK inhibitor - F (ID: At2g23430) | 5’- TATCGACGGGGTACGAAGAG -3’ |
| CDK inhibitor -R | 5’- CTAATGGCTTCTCCTTCTCG -3’ |
| P5C1- F (ID: At2g39800) | 5’- CAACCATGAGTACTGTGCCAA -3’ |
| P5C1-R | 5’- CCTCTCATTATCCATCTCGTT -3’ |
| Gsh1 – F (ID: Z29490) | 5’- GCTTTCTGGGTGGGTTTATTA -3’ |
| Gsh1-R | 5’- CTCCATTGTACATCTCCAAG -3’ |
| Gsh2- F (ID: X83411.1) | 5’- TTGTCAAGCAGGCTATCGAA -3’ |
| Gsh2-R | 5’- TCTTCGTGCGCATTAGATAAC -3’ |
| bHLH129- F (ID:AT2G43140.2) | 5’- GGGACAACTCGTCTTCTCAT-3’ |
| bHLH129-R | 5’- CAACATATCTGCGTAGCTCG -3’ |
| AKS2 bHLH128 - F (ID:AT1G05805.1) | 5’- TAGGGTTTACCGTGACTAGG -3’ |
| AKS2 bHLH128 -R | 5’- TACCAGCATGTCTGAATAGC -3’ |
| ATMYB60- F (ID**:**AT1G08810.1) | 5’- AAACATTTCCCGCCTTCTTGA -3’ |
| ATMYB60-R | 5’- CTCAATAAATGTCAATGGTGG -3’ |
| MYB44- F (ID:AT5G67300.1) | 5’- TTGTGCTACCGCTTCCTATC -3 |
| MYB44-R | 5’- CGTTGCATCTCCGTCATGTA -3’ |
