# Supplementary figures and images for: A Cyclin Dependent Kinase Regulatory Subunit (CKS) Gene of Pigeonpea Imparts Abiotic Stress Tolerance and Regulates Plant Growth and Development in Arabidopsis
Source: Front Plant Sci. 2017 Feb 10;8:165. doi: 10.3389/fpls.2017.00165 (PMC5301084; doi:10.3389/fpls.2017.00165)

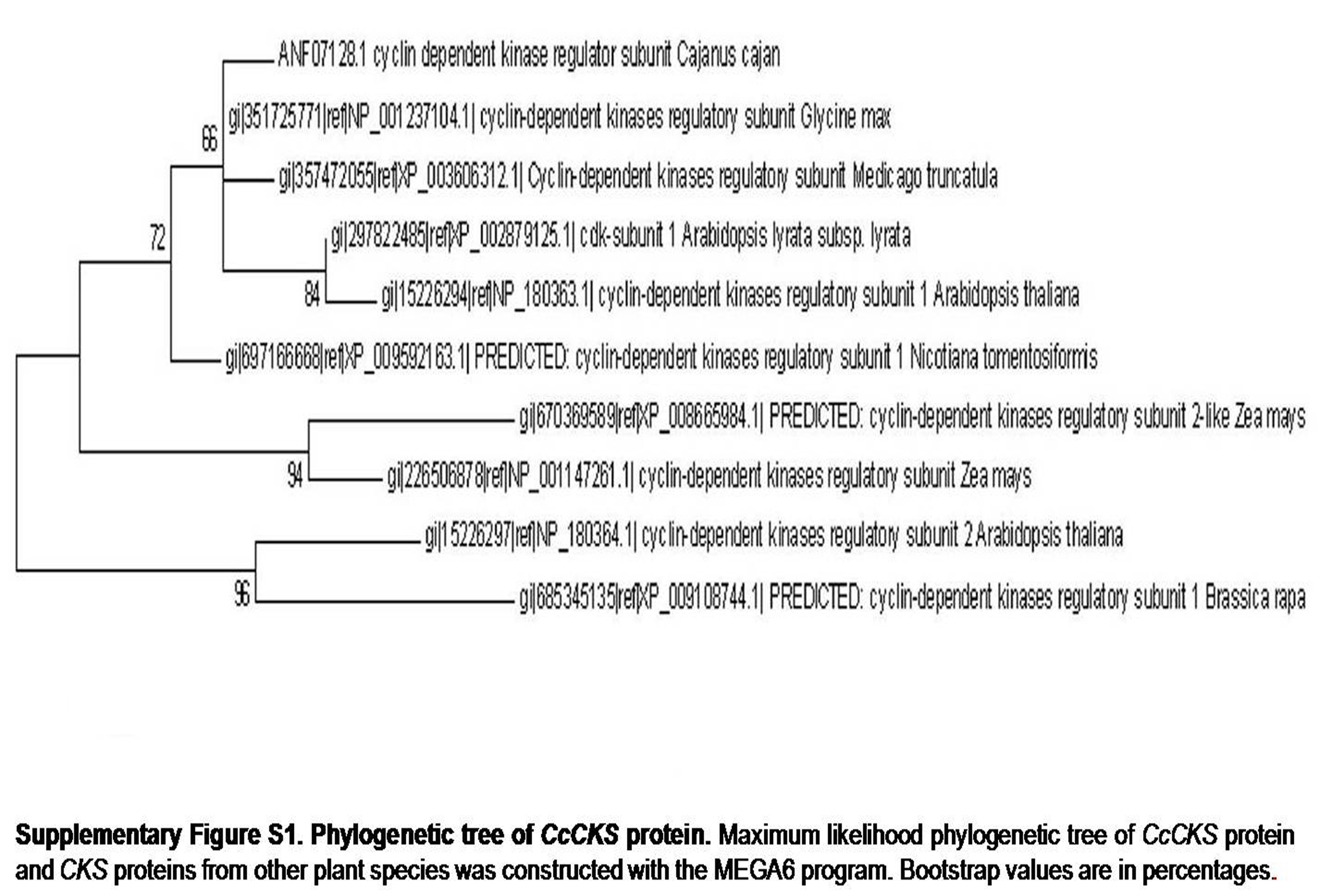

Supplement: Supplementary Figure S1 — Phylogenetic tree of CcCKS protein. [file Image1.JPEG]

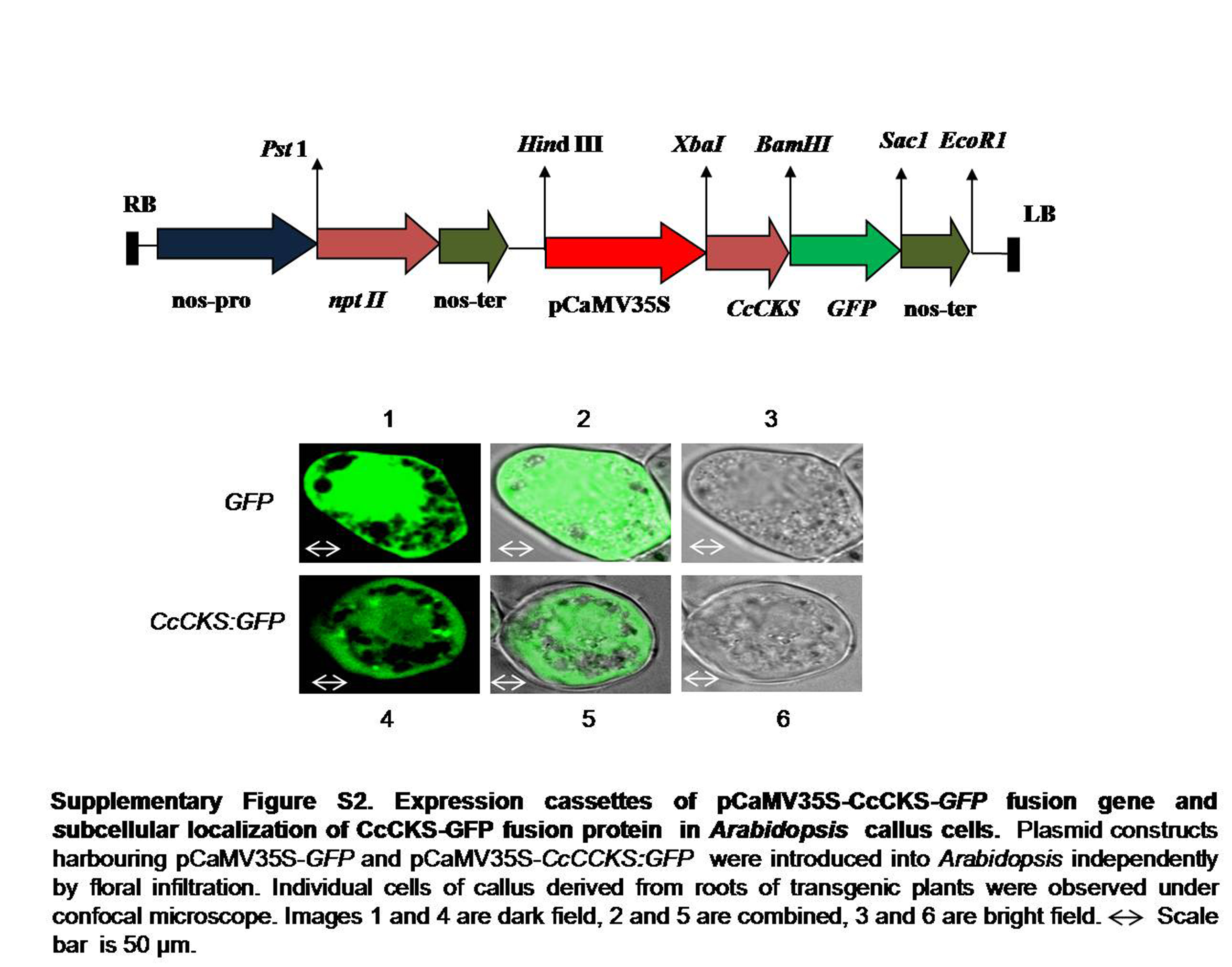

Supplement: Supplementary Figure S2 — Expression cassettes of pCaMV35S-CcCKS-GFP fusion gene and subcellular localization of CcCKS-GFP fusion protein in Arabidopsis callus cells. [file Image2.JPEG]

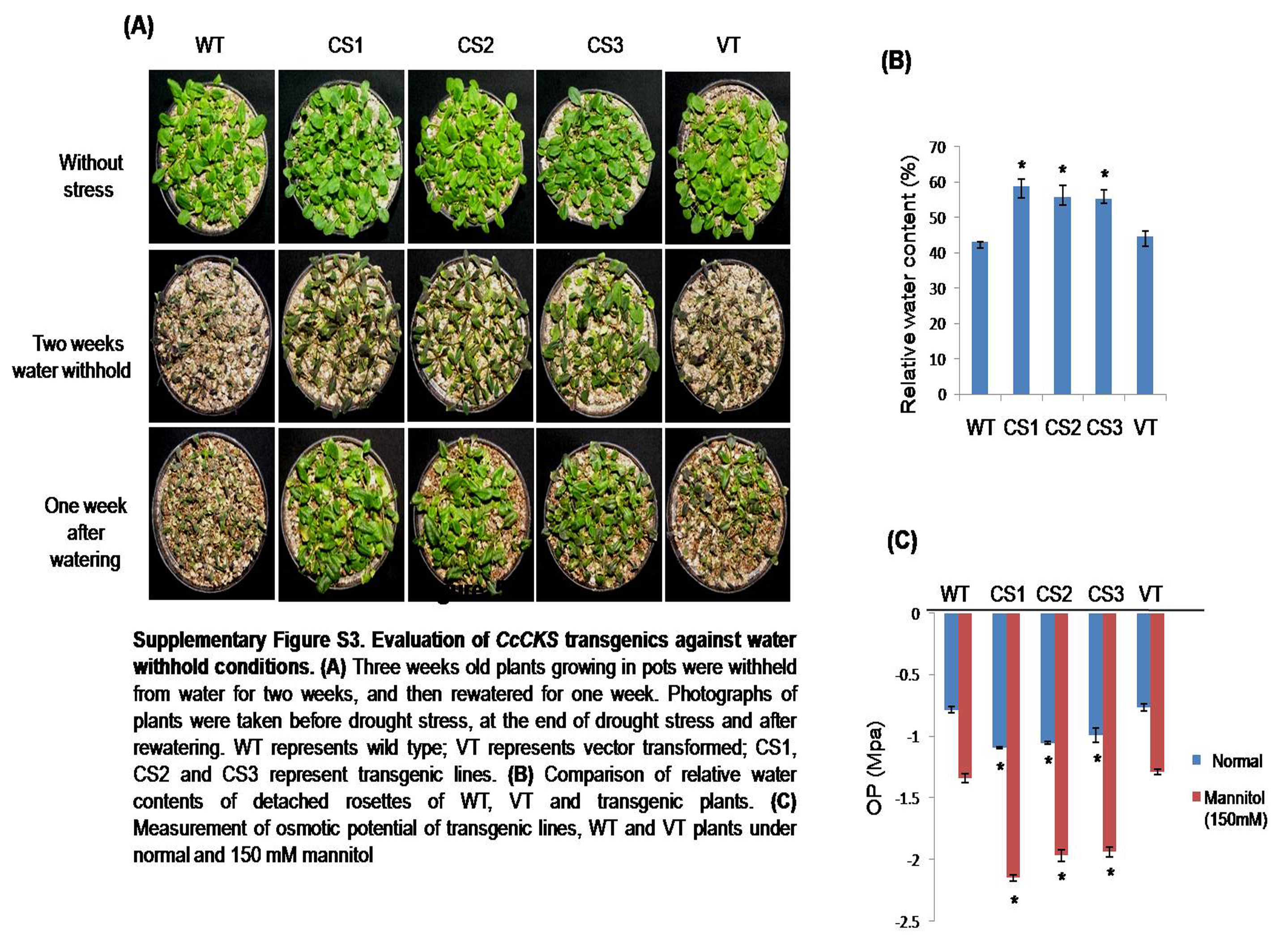

Supplement: Supplementary Figure S3 — Evaluation of CcCKS transgenics against water withhold conditions. (A) Three weeks old plants growing in pots were withheld from water for two weeks, and then rewatered for one week. Photographs of plants were taken before drought stress, at the end of drought stress and after rewatering. WT represents wild type; VI represents vector transformed; CS1, CS2 and CS3 represent transgenic lines. (B) Comparison of relative water contents of detached rosettes of WT, VI and transgenic plants. (C) Measurement of osmotic potential of transgenic lines, WT and VT plants under normal and 150 mM mannitol. [file Image3.JPEG]

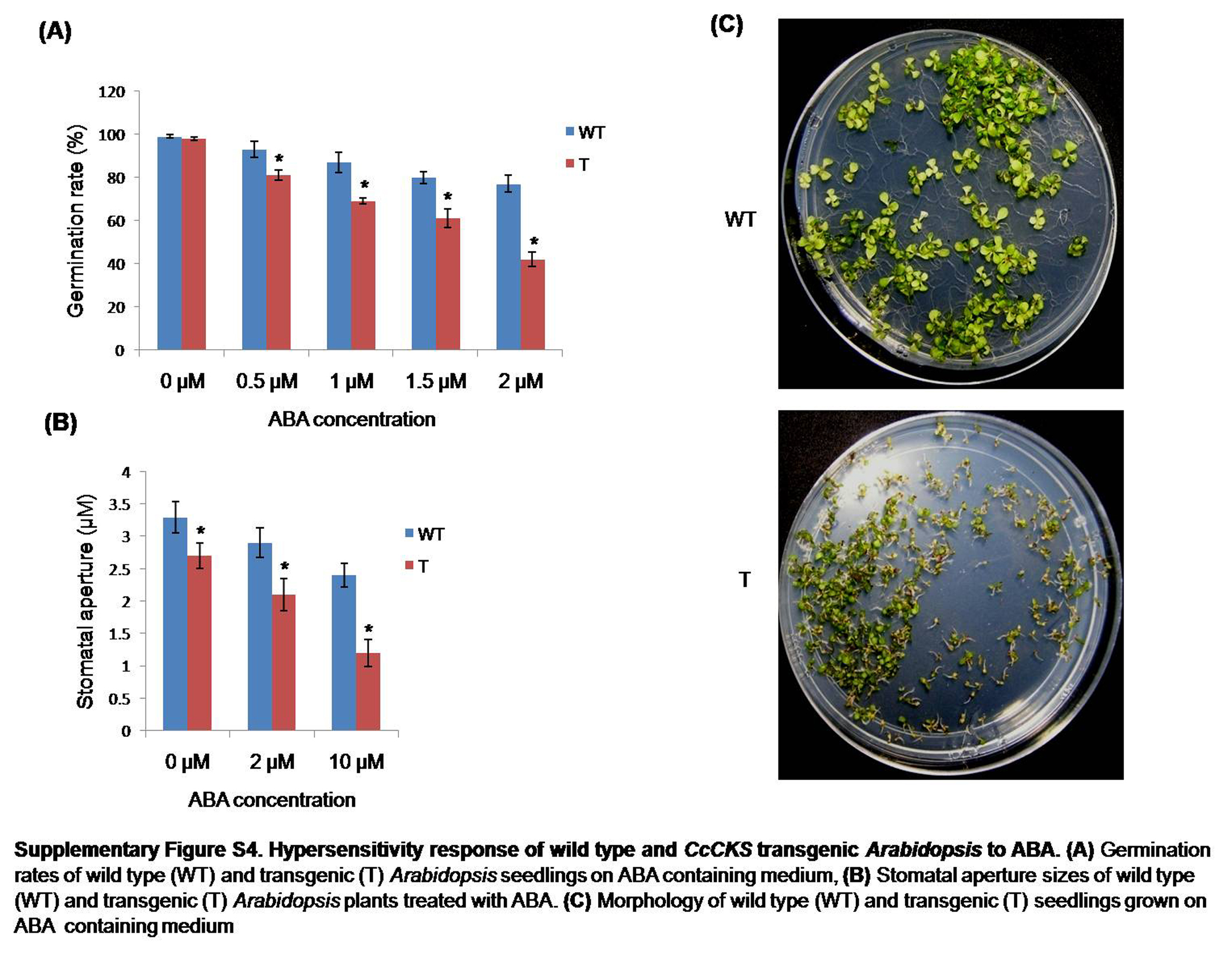

Supplement: Supplementary Figure S4 — Hypersensitivity response of wild type and CcCKS transgenic Arabidopsis to ABA. (A) Germination rates of wild type (WT) and transgenic (T) Arabidopsis seedlings on ABA containing medium, (B) Stomatal aperture sizes of wild type (WT) and transgenic (T) Arabidopsis plants treated with ABA. (C) Morphology of wild type (WT) and transgenic (T) seedlings grown on ABA containing medium. [file Image4.JPEG]

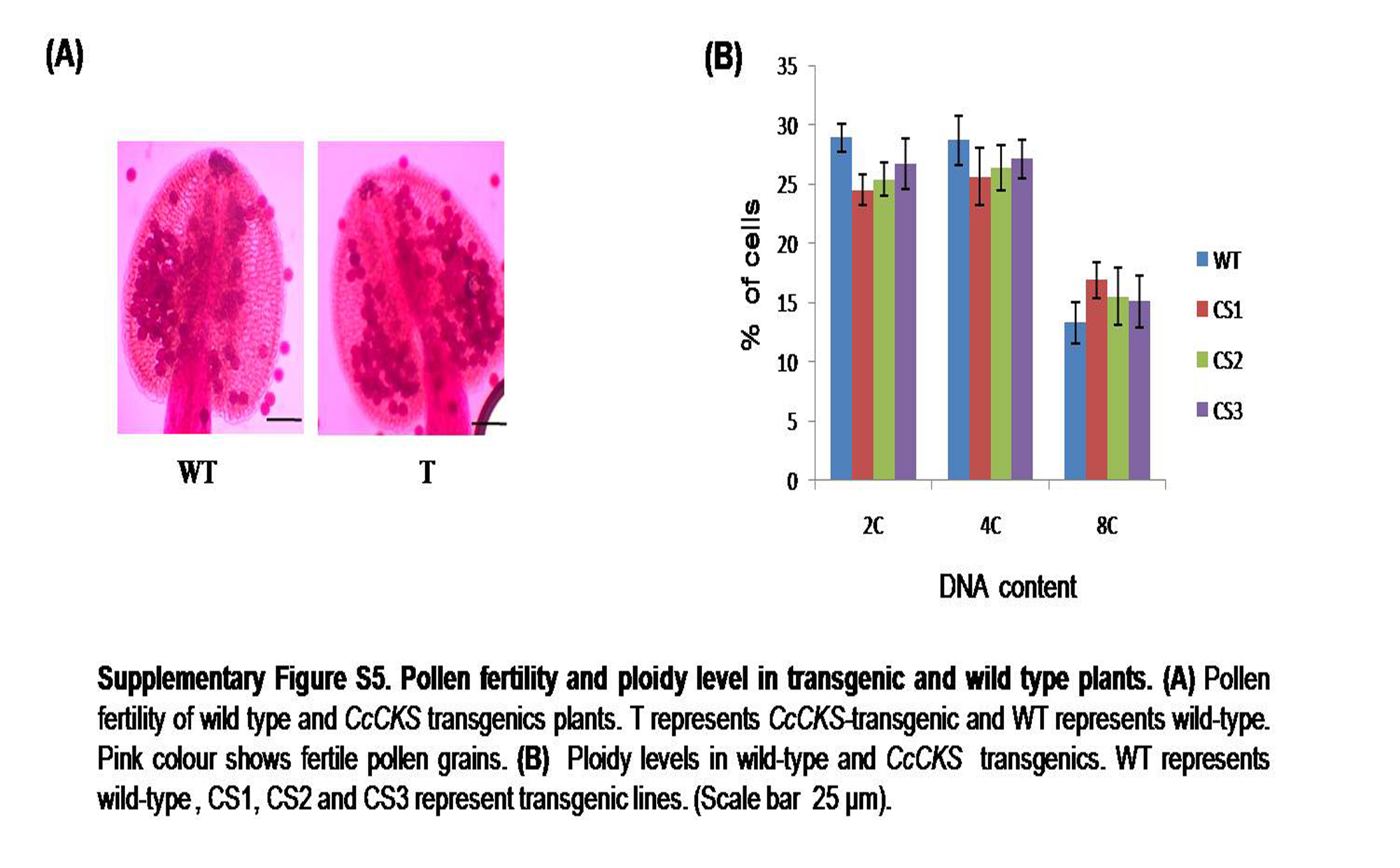

Supplement: Supplementary Figure S5 — Pollen fertility and ploidy level in transgenic and wild type plants. (A) Pollen fertility of wild type and CcCKS transgenics plants. T represents CcCKS-transgenic and WT represents wild-type. Pink color shows fertile pollen grains. (B) Ploidy levels in wild-type and CcCKS transgenics. WT represents wild-type, CS1, CS2 and CS3 represent transgenic lines. (Scale bar 25μm). [file Image5.JPEG]
